# Target Identification of *Mycobacterium tuberculosis* Phenotypic Hits Using a Concerted Chemogenomic, Biophysical, and Structural Approach

**DOI:** 10.3389/fphar.2017.00681

**Published:** 2017-09-26

**Authors:** Grace Mugumbate, Vitor Mendes, Michal Blaszczyk, Mohamad Sabbah, George Papadatos, Joel Lelievre, Lluis Ballell, David Barros, Chris Abell, Tom L. Blundell, John P. Overington

**Affiliations:** ^1^European Molecular Biology Laboratory, European Bioinformatics Institute, Cambridge, United Kingdom; ^2^Department of Biochemistry, University of Cambridge, Cambridge, United Kingdom; ^3^Department of Chemistry, University of Cambridge, Cambridge, United Kingdom; ^4^Diseases of the Developing World, GlaxoSmithKline, Madrid, Spain; ^5^Medicines Discovery Catapult, Alderley Edge, United Kingdom

**Keywords:** *Mycobacterium tuberculosis*, phenotypic hits, target identification, drug resistance, EthR, InhA

## Abstract

Mycobacterium phenotypic hits are a good reservoir for new chemotypes for the treatment of tuberculosis. However, the absence of defined molecular targets and modes of action could lead to failure in drug development. Therefore, a combination of ligand-based and structure-based chemogenomic approaches followed by biophysical and biochemical validation have been used to identify targets for *Mycobacterium tuberculosis* phenotypic hits. Our approach identified EthR and InhA as targets for several hits, with some showing dual activity against these proteins. From the 35 predicted EthR inhibitors, eight exhibited an IC_50_ below 50 μM against *M. tuberculosis* EthR and three were confirmed to be also simultaneously active against InhA. Further hit validation was performed using X-ray crystallography yielding eight new crystal structures of EthR inhibitors. Although the EthR inhibitors attain their activity against *M. tuberculosis* by hitting yet undefined targets, these results provide new lead compounds that could be further developed to be used to potentiate the effect of EthA activated pro-drugs, such as ethionamide, thus enhancing their bactericidal effect.

## Introduction

The pursuit of new and alternative drugs for the treatment of tuberculosis has led to phenotypic screens taking center stage in identifying novel and effective drug candidates (Lechartier et al., 2014). This screening effort has resulted in thousands of bioactive compounds released into the public databases, by researchers. The open access ChEMBL database (Gaulton et al., 2012, 2016) houses (as of February 2017) more than 16 datasets containing phenotypic hits for Neglected Tropical Diseases (https://www.ebi.ac.uk/chemblntd). These include the GSK TCAMS dataset containing about 776 phenotypic compounds that have shown activity against *Mycobacterium bovis* (IC_50_ < 10 μM), and have exhibited low hepatotoxicity levels (Ballell et al., 2013) in whole-cell screening assays. In particular, 177 of these compounds were found to be non-toxic for HepG2 cells [(HepG2 IC50/MIC) > 50] and have demonstrated the ability to also highly inhibit the growth of the related *Mycobacterium tuberculosis* (MIC against H37Rv of < 10 μM), the main causative agent of tuberculosis, a disease affecting about 9 million people every year.

The success of phenotypic hits, in preclinical and clinical drug development depends to a greater extent, on the knowledge of their mechanism of action. However, the protein targets for most of the reported phenotypic hits are still to be identified. Experimentally identifying targets for all the phenotypic hits involves a broad range of approaches including genetic, proteomic, and transcriptional profiling, and more direct chemical-proteomic methods (Hart, 2005). It is therefore advantageous, after phenotypic screening to use computational methods that can predict likely protein targets of a given active small molecule, providing target-ligand pairs as starting points, thereby narrowing the search for the target proteins (Szardenings et al., 2004; Jenkins et al., 2006; Rebollo-Lopez et al., 2015).

In chemogenomics predictive approaches, targets are proposed by considering the chemical structural features of the active compounds (Jenkins et al., 2006) available in databases such as ChEMBL and PubChem BioAssay and comparing these to features of known ligands of a set of targets. Some of these approaches include ligand-based 2D chemical similarity assessment, cluster analysis and the use of 3D descriptors when the orphan compound has low similarity to all database molecules (Jenkins et al., 2006; Bender et al., 2007). In addition, machine-learning methods are also being used to extract targets and their associated ligands automatically from target-ligand information stored in multiple-target models. The multiple-category Laplacian-corrected Naïve Bayesian Classifiers (MCNBC) trained on extended-connectivity fingerprint of 964 targets classes in the WOMBAT database were originally described by Nidhi et al. (2006) and have been previously used (Ekins et al., 2013; Martínez-Jiménez et al., 2013) to identify targets for the TCAMS anti-TB phenotypic hits. However, experimental confirmation of the predicted targets for these compounds is limited and in most instances, still to be reported.

The enoyl-acyl carrier protein reductase (InhA) is a well-known anti-TB target involved in the biosynthesis of mycolic acids and is the target of first- and second-line TB drugs, isoniazid, and ethionamide respectively. The two drugs require metabolic activation inside *M. tuberculosis* cells by specific proteins; the heme enzyme catalase peroxidase (KatG) for isoniazid, and FAD-containing monooxygenase (EthA) for ethionamide; interestingly much of the clinically observed resistance is associated with the drug activation mechanisms (Takayama et al., 1972; Timmins and Deretic, 2006). Studies of the resistance mechanism of ethionamide revealed that an increase in the expression of HTH-Transcriptional regulator, EthR, reduces transcription and the level of active EthA protein, and results in resistance by *M. tuberculosis* (DeBarber et al., 2000; Dover et al., 2004). The discovery of this transcriptional regulator led to the notion of controlling EthA expression levels by targeting EthR and thus boosting the bactericidal effect of ethionamide (Frenois et al., 2004; Willand et al., 2009). Therefore, identifying compounds that bind to EthR and prevent the repression of EthA would not only circumvent some of the resistance mechanisms but also reduce the required dosage and therefore the toxicity associated with this drug. In this work, we report for the first time, inhibitors of two *M. tuberculosis* targets, (EthR and InhA), identified using predictive *in silico* methods and definitively confirmed through biophysical methods, biochemical assays, and X-ray crystallography.

## Methods

### Identification of targets of anti-TB phenotypic hits

In previous work, we reported a detailed description of the target prediction protocol (Mugumbate et al., 2015) comprising of two ligand-based and one structure-based approach to identify targets for the 776 TCAMS-TB compounds (Ballell et al., 2013) retrieved from the ChEMBL database (https://www.ebi.ac.uk/chembl/). The same protocol and models were used in this work to identify targets for further anti-TB phenotypic hit compounds; hence a brief description of the ligand-based method is presented here. The dataset of anti-TB compounds was prepared using a protocol in Pipeline Pilot version 8.5 (Warr, 2012), by generating 2D coordinates for each compound, and standardizing stereochemistry, and formal charges for common functional groups. In addition, salts and single atom were removed.

### Ligand-based target identification

A dataset containing 698,401 human and bacterial target-ligand pairs was extracted from ChEMBL version 17 (http://www.ebi.ac.uk/ChEMBL). The dataset contained targets with an annotated ChEMBL confidence score (CS) ranging from 7 to 9, indicating that the target—ligand pairs have unambiguously defined protein complexes or single proteins. Filtering the dataset to collect targets with 10 or more annotated ligands that exhibited IC_50_, EC_50_, or K_i_ values ≤ 10 μM or inhibition ≥ 50% against their respective targets resulted in a total of 2,257 unique human and bacterial proteins,. Amongst these were 50 proteins belonging to the pathogenic bacterial species, *M. tuberculosis*. Human proteins were included to enable identification of potential host off-targets for the anti-tuberculosis compounds. MCNBCs were generated by training the protein identifiers on the structural features of their compounds, defined by their extended-connectivity fingerprint of diameter 6 (ECFP_6) (Rogers and Hahn, 2010) in Pipeline Pilot.

We determined the robustness of our models by a guaranteed random selection of compounds for both the training and test sets and minimized bias by presenting the model with a test set of previously unseen compounds using *two* methods (Supporting Information). In both instances the models displayed 75–90% accuracy, quantified by the percentage of compounds with correctly assigned targets reported in ranked positions 1–5. During the production stage, a model MCNBC trained on 695,902 target-ligand pairs was used to identify targets for all 776 phenotypic hits. We retained pairs of targets and compounds with positive Bayesian (NB)-scores and Z-scores ≥ 1.5. The Bayesian Score is defined as the sum (P_*total*_) of the logarithm of Laplacian-corrected Bayes rule of conditional probability [P(A|Fi)] for each fingerprint feature of a compound **(12, 21)**. The Z-score distinguishes the compound scores for a particular target from the influence of the background noise. The score was obtained by predicting targets for > 1,200,000 compounds in ChEMBL version 17 database and generated the background information by calculate mean NB scores (μ) and standard deviation (σ) for each target. For each predicted target, standard scores (Z-score) were calculated from a statistical analysis of the NB scores for each compound: Z-score = (X − μ)/σ where, X is the NB score of a target for a compound. We have similarly generated Bayesian models using ChEMBL versions 20 and 21 data, however here we report the results obtained using ChEMBL 17 based models used at the time of the study.

These predictions were complemented by utilizing a second and independent ligand-based approach, The Similarity Ensemble Approach (SEA), which exploits chemical similarity between two sets of ligands, was used to study the pharmacological relationships between drugs (Keiser et al., 2009; Lounkine et al., 2012) and to predict targets for all 776 GSK phenotypic hits. At the time of this study the latest version of SEA utilized ChEMBL version 16 data and extended-connectivity fingerprint of diameter 4 (ECFP_4) fingerprints. To validate the method, the tool was used to predict targets for TB drugs of known modes of action where the bioactivity data are not found in ChEMBL version 16. We selected predicted *M. tuberculosis* protein targets from target-ligand pairs displaying expectation (E) values < 10^−1^. In general, the E-value describes the significance of similarity between an orphan compound and a set of ligands and is a good indicator for the most likely targets (Keiser et al., 2007). A cut-off of 10^−1^ guaranteed selection of all possible target-ligand pairs and was suitable for our purposes.

### Structure-based target identification

The MCNBC and SEA methods provided a set of potential predicted targets, from which targets to use in structure-based target identification were selected. The Internal Coordinate Mechanism (ICM) method developed by Molsoft L.L.C (Totrov and Abagyan, 1997) was used to estimate the binding affinities of the anti-TB phenotypic hits to two predicted *M. tuberculosis* targets, EthR and InhA, based on the ICM scoring function, and available structures in wwPDB. The scoring function takes into consideration the energy changes due to protein-ligand binding and is defined as:

$$\begin{array}{l} \Delta \text{G}=\Delta {\text{E}}_{\text{IntFF}}+\text{T}\Delta {\text{S}}_{\text{Tor}}+\alpha_{1}\Delta {\text{E}}_{\text{HBond}}+\alpha_{2}\Delta {\text{E}}_{\text{HBDesol}}\\ +\;\alpha_{3}\Delta {\text{E}}_{\text{SolEl}}+\alpha_{4}\Delta {\text{E}}_{\text{HPhob}}+\alpha_{5}{\text{Q}}_{\text{Size}} \end{array}$$

where: ΔE_IntFF_ is change in van der Waals interactions of ligand and receptor and the internal force-field energy of the ligand, TΔS_Tor_ is the change in free energy due to conformational entropy and weighted (α1 − α5), ΔE_HBond_ is the hydrogen bond term, ΔE_HBDesol_ accounts for the disruption of hydrogen bonds with solvent, ΔE_SolEl_ is the solvation electrostatic energy change upon binding, ΔE_HPhob_ is the hydrophobic free energy gain and Q_Size_ is the ligand size correction term.

### Preparation of the EthR and InhA proteins for docking

We extracted the crystal structure of *M. tuberculosis* EthR bound to the inhibitor BDM31369 (PDB code: 3Q0V, resolution 1.95 Å) from the Protein DataBank in Europe (PDBe). The structure is one of the EthR structures containing synthetic inhibitors that occupy the allosteric pocket. The structure is dimeric and consists of chain A with 188 ordered residues and chain B containing 194 ordered residues, bound to the inhibitor BDM31369 (PDBE code; LL4). Some of the key residues interacting with the ligand include L87, I107, F110, M142, Y148, T149, V152, N176, and W207. Based on the structure validation from PDBe, chain A with higher quality backbone and side chain geometry percentiles, was used in our docking calculations. Using standard ICM-docking (Neves et al., 2012) receptor preparation tools the EthR structure was prepared by deleting all water molecules, adding and optimizing hydrogen atoms, adding missing heavy atoms, and adjusting amide groups. The “setup receptor” tool was used to generate receptor maps that cover the entire binding pocket of EthR using the crystallized ligand to determine the grid center, a grid size of 0.5 Å, and grid dimensions (x, y, z) of 51, 43, and 40 Å. The performance of the docking procedure was evaluated by redocking the co-crystallized ligand at thoroughness value of 1, which regenerated the crystal conformation at root mean square deviation (RMSD) of 1.81 Å between the Cα atoms of the docked and the co-crystallized ligands. This proved that the software regenerated the crystal structure conformation and accurately docked the ligand.

Similarly, we extracted the 3D coordinates of an InhA structure in complex with the inhibitor PT92 (PDBe code: 4OHU, resolution of 1.6 Å) with the best structure-quality score from the PDBe. In the presence of the co-factor, NAD, the inhibitor occupies the substrate-binding pocket and primarily interact with residues L218, A157, M199, M161, Y158, and the nicotinamide moiety of NAD. The structure is tetrameric each chain with 289 residues and chains A, B, and C had percentiles of 100%. Chain A was used in the docking calculations. ICM molecules were generated using the ICM-docking receptor tools to generate grid maps of dimensions (x, y, z) 49, 46, and 44 Å, using grid step of 0.50 Å. After preparation of the crystal ligand, it was re-docked into the binding site and its conformation was regenerated at root mean square deviation of 0.86 Å between the Cα atoms of the docked and co-crystal ligands.

### Preparation of small molecules and docking

Three-dimensional coordinates of 776 anti-TB compounds were generated using a Pipeline Pilot protocol. The molecules were imported into ICM (Totrov and Abagyan, 1997), amide bonds were fixed, hydrogen atoms were built and the structures were converted into ICM molecules. The compounds were docked into EthR and InhA using thoroughness/effort value of 2 and other default ICM parameters. The ICM scores were standardized by calculating the ligand efficiency indices (LEI), defined as the ratio between the ICM score and the number of heavy atoms for each docked molecule (Abad-Zapatero et al., 2010). Compounds with LEI ≥ 1.0 were retained for further analysis for each target protein.

### Compounds

All anti-tuberculosis phenotypic compounds used in this work were provided by GSK TCAMS project, Spain, as 10 mM DMSO solutions.

### Bacterial strains and cloning

*inhA and ethR* genes were amplified from chromosomal DNA of *M. tuberculosis* H37Rv DNA acquired from ATCC (ATCC25618D-2) and cloned in a pET28a vector (Novagen), modified with an N-terminal 6xHis-SUMO tag between BamHI/HindIII sites and in pHAT5 vector (Peranen et al., 1996) between BamHI/EcoRI sites, respectively. The resulting constructs were confirmed by DNA sequencing and transformed into *E. coli* BL21(DE3) strain (Invitrogen).

### Recombinant expression and protein purification

*E. coli* BL21(DE3) transformed with *inhA* and *ethR* containing plasmids cells were grown to mid-exponential growth phase (OD_610_ = 0.8) in LB media (Invitrogen) containing 30 mg.l^−1^ kanamycin (*inhA*) or 100 mg.l^−1^ Ampicilin (*ethR*) at 37°C. Isopropyl β–D-1-thiogalactopyranoside (IPTG) was then added at a final concentration of 0.5 mM to induce gene expression and the temperature was lowered to 18°C. EthR purification followed the protocol previously described by us (Surade et al., 2014). For InhA cells were harvested 18–20 h later by centrifugation and re-suspended in 50 mM HEPES pH 7.5, 0.5 M NaCl, 10% glycerol (w/v) and 20 mM imidazole with protease inhibitor tablets (Roche), DNAseI and 5 mM MgCl_2_. Cells were lysed by sonication and the cell lysate was centrifuged at 27,000 g for 30 min to remove cell debris. Recombinant InhA was purified with a HiTrap IMAC Sepharose FF column (GE-Healthcare), equilibrated with 50 mM HEPES pH 7.5, 0.5 M NaCl, 10% glycerol (w/v), and 20 mM imidazole. Elution was performed in the same buffer with 500 mM imidazole. Imidazole was removed by dialysis into 50 mM HEPES pH 7.5, 0.5 M NaCl and 10% glycerol (w/v) and SUMO tag was cleaved overnight at 4°C by adding Ulp1 Protease at 1:100 ratio. SUMO tag, Ulp1 protease and uncleaved SUMO-InhA were removed with a HiTrap IMAC Sepharose FF column (GE-Healthcare), equilibrated with 50 mM HEPES pH 7.5, 0.5 M NaCl, 10% glycerol (w/v), and 20 mM imidazole. Flow through containing InhA was collected, concentrated and loaded in a Superdex 200 column equilibrated with 50 mM HEPES pH 7.5, 150 mM NaCl, and 10% glycerol (w/v). Fraction purity was determined by SDS-PAGE. The purest fractions were pooled, concentrated to ~12 mg.ml^−1^, flash frozen in liquid nitrogen and stored at −80°C. Protein concentration was determined using the Bradford method.

### Enzymatic assays (InhA)

InhA activity was assessed by a spectrophotometric assay that followed the oxidation of NADH at λ = 340 nm in the presence of 2-trans-octanoyl-CoA. Compounds were dissolved in 100% DMSO. The enzyme reaction contains 30 mM PIPES pH 7.5, 50 mM NaCl, 1% (v/v) DMSO and 0.1 mM EDTA. InhA (100 nM) was pre-incubated for 10 min at room temperature with 0.25 mM NADH and compounds at a final concentration of 100 μM in 150 μl reaction volume. The reaction is started by the addition of 2-trans-octanoyl-CoA at a final concentration of 1.5 mM, prepared as described previously (He et al., 2006). The reactions were followed for 20 min using a spectrophotometer plate reader (CLARIOstar—BMG LABTECH). Inhibition percentage was calculated based on the initial rates of reaction. All experiments were performed in triplicate.

### Surface plasmon ressonance assay (EthR)

The surface plasmon resonance (SPR) assay was performed on a BIAcore T100 with a protocol similar to what was previously described (Surade et al., 2014). Briefly, *M. tuberculosis ethA* promoter DNA (106 bp) was amplified with 5′-CGGTCATGGATCCACGCTATCAAC-3′ and 5′-biotin-CTGACTGGCCGCGGAGGTGGT-3′ and immobilized in a SA chip (GE Healthcare). A control DNA fragment from pUC19 (113 bp) was amplified in a similar fashion. Biotinylated DNA (promoter and control) was then flowed over different channels and similar levels of resonance units (RUs) were achieved. For the initial screening a single run was performed for each compound. A 2 μM EthR solution was then incubated individually with each of the compounds at 100 μM, in 2 mM MgCl_2_, 10 mM TRIS/HCL pH 7.5, 200 mM NaCl, 0.1 mM EDTA, and 2% DMSO and flowed over the chip at 20 μl/min for 120 s. For dissociation 150 s were given using the same flow rate. Binding was determined by subtracting the response of the control channel from the experimental channel at steady-state. Regeneration of the chip was performed between samples by flowing 0.03% SDS in running buffer for 60 s at 20 μl/min. To calculate IC_50_, EthR-DNA-binding response, in the presence of compounds, was measured at several compound concentrations between (0.78 and 100 μM). Data were analyzed in Prism 5 (Graphpad software) and IC_50_ were calculated using a non-linear regression model. Dose response experiments were performed in triplicate.

### Crystallization

EthR crystallization and compound soaking of crystals was carried out as previously described (Surade et al., 2014). An exception to this was compound **3** where co-crystallization was required. For this purpose EthR at 20 mg.ml^−1^ was incubated for 30 min on ice with 1 mM compound and 10% DMSO. Co-crystallization was performed at 18°C, using the sitting drop method and several commercial crystallization screens were tested (PEG I, (Qiagen); JCSG-plus, PACT, wizard classic I&II and III&IV (Molecular Dimensions). The highest diffracting condition for compound 3 was found in PACT crystallization screen solution E12 (0.2 M sodium malonate dibasic and 20% PEG 3350) using 0.3 μl EthR-compound 3 solution and 0.6 μl reservoir solution. All the other compounds that were co-crystallized with EthR did not yield crystals or the crystals diffracted only at low resolution. A cryogenic solution was prepared by adding ethylene glycol up to 25% v/v to mother liquor. Crystals were briefly transferred to this solution, flash frozen in liquid nitrogen and stored for data collection.

### Data collection, structure solution, and refinement

All data sets were collected at stations I02, and I04, at Diamond Light Source (Oxford, UK). Data collection and refinement statistics are summarized in Table S1.

Diffraction data were indexed, integrated, and reduced using autoPROC from Global Phasing Limited (Vonrhein et al., 2011). Molecular replacement was performed with Phaser (McCoy et al., 2007) using PDB structure 1T56 as a search model. Refinement was carried out iteratively with PHENIX (Adams et al., 2010) and Coot (Emsley et al., 2010), and ligand and water fitting was performed with Coot (Emsley et al., 2010).

## Results and discussion

### Predicted *M. tuberculosis* targets

**Figure 1:**
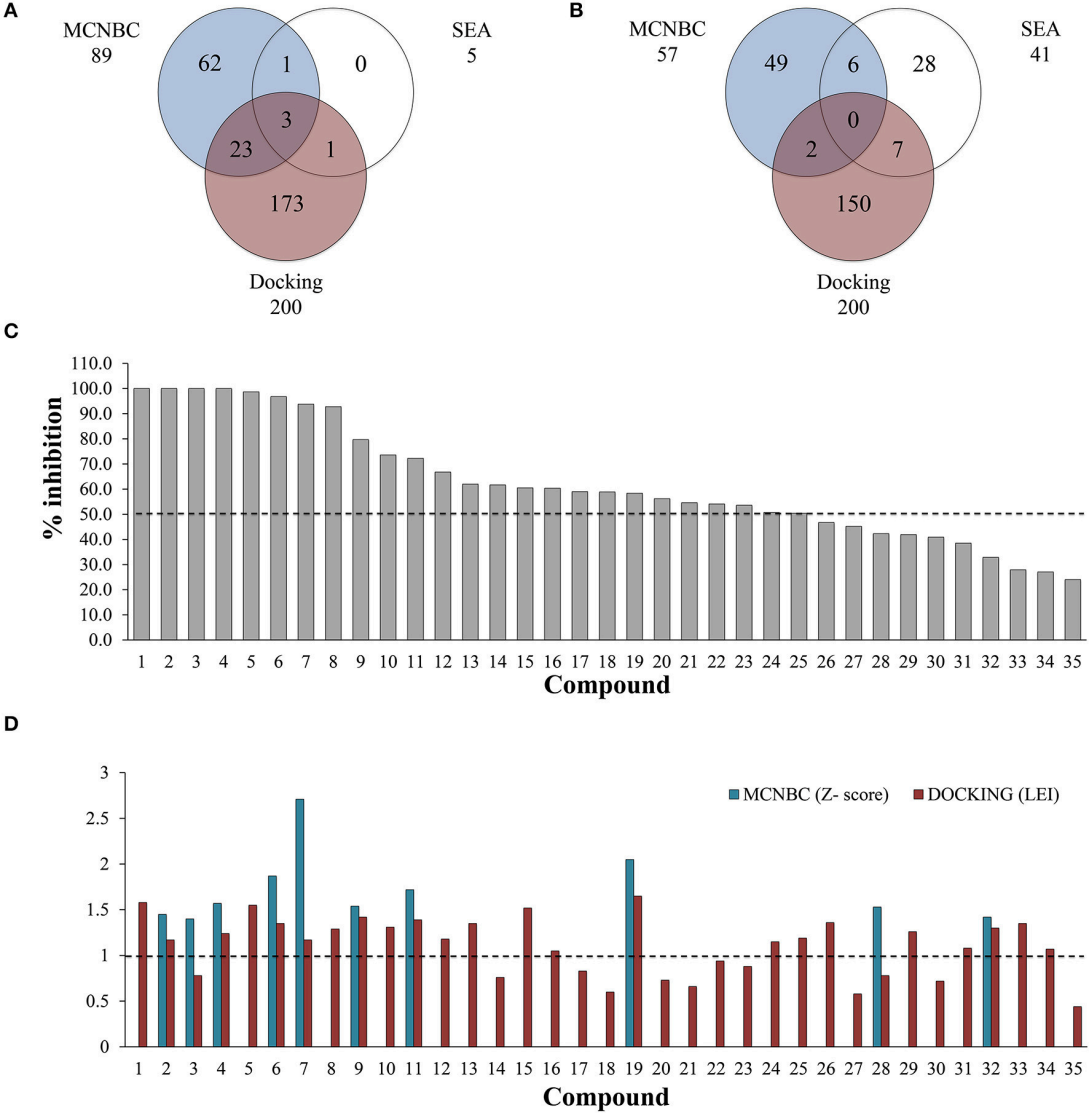
Number of ligands predicted to inhibit EthR **(A)** and InhA **(B)** using MCNBC, SEA, and docking calculations. A total of 28 and 15 compounds were jointly identified by the three methods for EthR and InhA respectively. **(C)** Out of 35 compounds tested for against EthR using Surface Plasmon Resonance (SPR), 25 exhibited at least 50% inhibition of the enzyme, with four compounds showing 100% inhibition at 100 μM. **(D)** The complementary effect of MCNBC and docking methods. The two methods successfully identified 19/25 inhibitors of EthR at Z-score and Ligand Efficiency Index cut-offs of 1 for MCNBC and Docking respectively (broken lines).

We initially predicted 1,462 human and bacterial potential targets for the 776 GSK anti-TB compounds using the Multiple Category Naive Bayesian Classifiers (MCNBC). Each potential target displayed positive Bayesian scores (NB) and standard deviation scores (Z-scores) ≥ 1.5 for respective ligands. In this predicted target set, 146 (10%) were bacterial proteins and 25 of these were *M. tuberculosis* proteins, which were assigned to 132 compounds. To obtain a wider coverage and possibly identify novel targets, we mapped functional data and chemical bioactivity data of all predicted human and bacterial targets across their *M. tuberculosis* orthologs as reported in the OrthoMCL database (Chen et al., 2006). This increased the number of predicted *M. tuberculosis* targets to 137 (Figure 1) for 517 compounds, and accounted for 4,575 target-ligand pairs (Table S2A). Similarly, the second ligand-based method, SEA generated 36,607 target-ligand pairs for 1,346 proteins from all the organisms in ChEMBL version 16. After considering the *M. tuberculosis* orthologs in OrthoMCL, 110 targets were assigned to 428 compounds to give 1225 target-ligand pairs (Table S2B).

The predicted *M. tuberculosis* protein targets are involved in many known essential biological processes, with lipid metabolism having the highest number of predictions (Table 1). Although ~5% of the predicted protein targets for these compounds are established as essential for *M. tuberculosis* (Tables S2A,B), many others are not. Nevertheless, all compounds are predicted to hit multiple proteins with at least one essential protein per compound that could account for the observed compound activity in the phenotypic screen. Top predicted essential protein targets include many enzymes involved in lipid biosynthesis such as Pks13 (*Rv3800c*), a polyketide synthase involved in mycolic acid biosynthesis (Gavalda et al., 2009) and InhA (*Rv1484*) an anti-TB target of the approved drugs isoniazid and ethionamide, both part of the FASII system. Amongst the several non-essential proteins, is EthR (*Rv3855*) that is involved in ethionamide resistance (Tables S2A,B). For these reasons, we selected InhA and EthR to validate further the computational predictions. As aforementioned, targeting EthR presents a potential way to “boost” the anti-TB activity of ethionamide (Willand et al., 2009).

**Table 1:** Target prediction according to functional category.

**Functional category**	**Number of proteins**
DNA replication, recombination, and repair	10
Transcription	2
Ribosome and translation	7
Post-translational modification, protein degradation, and chaperones	10
Signal transduction	2
Detoxification and defense	5
Energy production	11
Cell envelope biogenesis	8
Nucleotide metabolism	13
Amino acid metabolism	10
Co-factor metabolism	7
Lipid Metabolism	33
Inorganic ion metabolism	3
Secondary metabolites biosynthesis and catabolism	15
General function prediction	18
Unknown function	7

### EthR and InhA inhibitors

#### Chemogenomic predictions

The mycobacterial HTH-transcriptional regulator (EthR) has been reported to be a facilitator for the resistance of the second-line tuberculosis drug, ethionamide. The protein was amongst the *M. tuberculosis* proteins with the most compound predictions having 90 assigned ligands using both MCNBC and SEA (Figure 1A). EthR binding tunnel is a hydrophobic cavity with multiple aromatic residues decorating its surface and a few charged residues making it a promiscuous binding site known to bind molecules with different properties (Willand et al., 2009, 2010; Flipo et al., 2012; Surade et al., 2014; Villemagne et al., 2014; Nikiforov et al., 2016, 2017). It also possesses multiple hotspots regions were fragments bind (Surade et al., 2014; Villemagne et al., 2014; Nikiforov et al., 2016, 2017). It is therefore not surprizing that this protein had a high number of predicted inhibitors as these characteristics make this site promiscuous. Extensive studies have already been performed on this protein. In excess of 20 crystal structures of EthR in complex with allosteric inhibitors have been reported. However, none of these have reached Phase I clinical trials, and therefore the hunt for inhibitors is still ongoing.

To complement the ligand-based predicted ligands for EthR, we used ICM dock (Neves et al., 2012) to dock all the 776 TCAMS compounds to a previously prepared crystal structure of *M. tuberculosis* EthR bound to an inhibitor BDM31369 (PDB code: 3Q0V). Most compounds docked into the long hydrophobic pocket of EthR and formed interactions with the side chains of W103, W207, and F110 residues and hydrogen bonds with N176 and N179. After ranking the compounds based on their calculated ligand efficiency index (LEI), 200 potential inhibitors displaying LEI greater than or equal to 1.0 were selected for further analysis. Three potential inhibitors of EthR were jointly predicted by the three complementary chemogenomic approaches, 23 compounds were identified by MCNBC and docking calculations whilst MCNBC/SEA and SEA/DOCKING each had one common compound (Figure 1A).

The clinical utility of the first-line anti-TB drug isoniazid (INH) against the enoyl acyl protein reductase InhA, is being challenged by the increasing drug resistance attributed to mutations or deletions in the active site of katG gene, which encodes catalase-peroxidase KatG, an enzyme responsible for the metabolic activation of INH (Vilcheze and Jacobs, 2014). For this reason, it is imperative to identify new drugs that target InhA but circumvent the KatG activation pathway. We used the chemogenomic methods MCNBC and SEA to synergistically allocate more than 80 as potential inhibitors of InhA. From the docking calculations 159 compounds displayed LEI ≥ 1.0 indicating favorable binding affinity to InhA (Figure 1B). Even though no compounds were jointly predicted by all three methods, about 15 compounds were commonly predicted by either docking and SEA, MCNBC, and docking or MCNBC and SEA approaches.

From the predicted EthR inhibitors, we selected a set of 26 compounds (**1–16**, **19**, **24–26**, **28–29**, and **31–34**), that were identified by one or more of the computational methods above, and for which physical samples were available for use in the *in vitro* assays. Approximately one third of these compounds displayed Z-scores ≥ 1.00 indicating similar structural features to those previously reported for EthR ligands in our training set. To investigate the ability of the chemogenomic methods to distinguish between true and false negatives our second set contained 9 compounds (**17–18**, **20–23**, **27**, **30**, and **35**) from the 776 GSK-TCAMS dataset, that fell outside the selection criteria and represent different molecular clusters. The target prediction scores for the 35 compounds are given in Table S3. About 51% (17 compounds) of this set was predicted to inhibit both EthR and InhA using both ligand-based and structure-based methods. Hence, *in vitro* experiments were also performed to confirm the activities of the 35 compounds against InhA.

### Validation of EthR inhibitors

**Figure 2:**
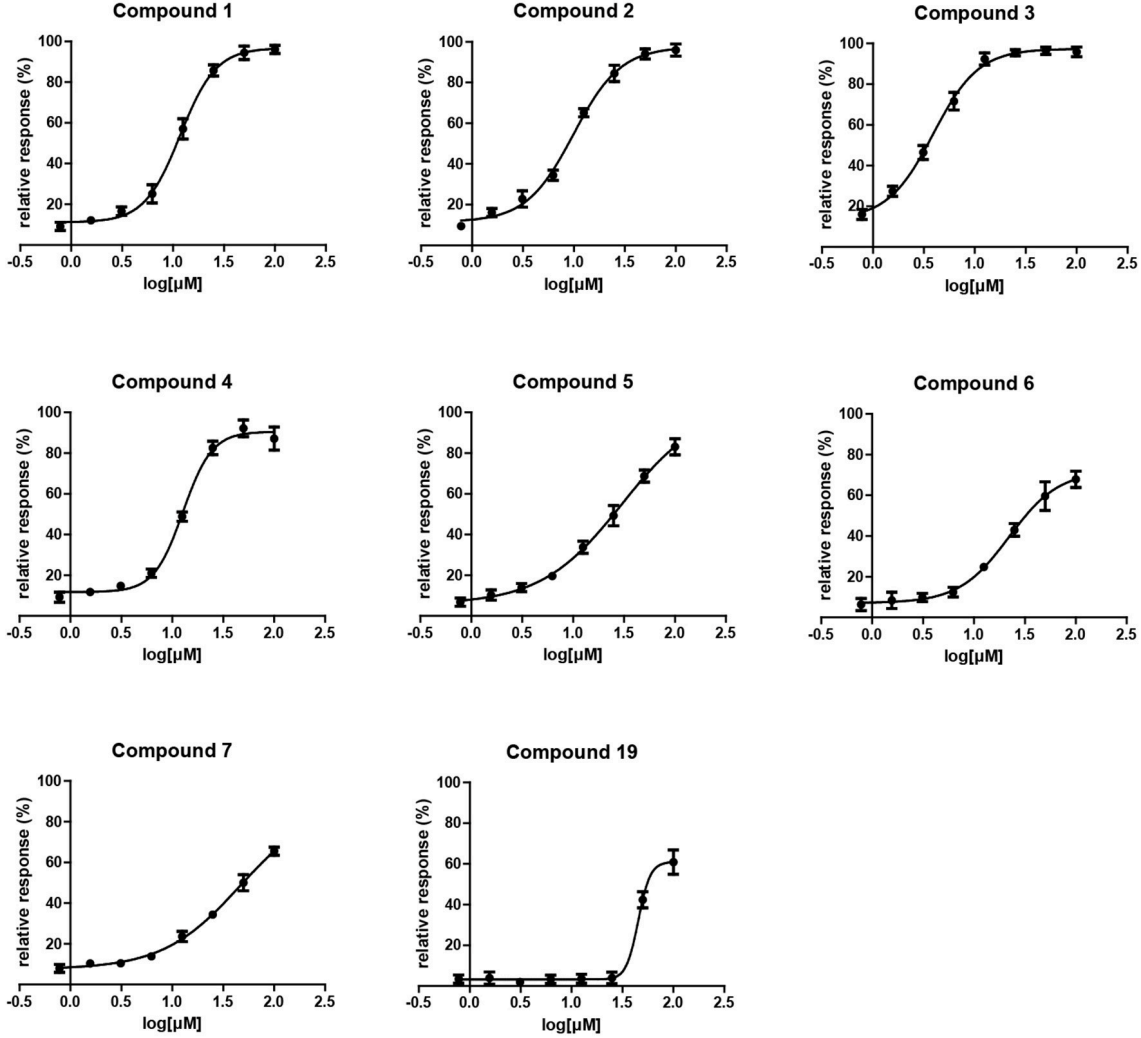
Surface plasmon resonance dose response curves for EthR inhibitors.

**Figure 3:**
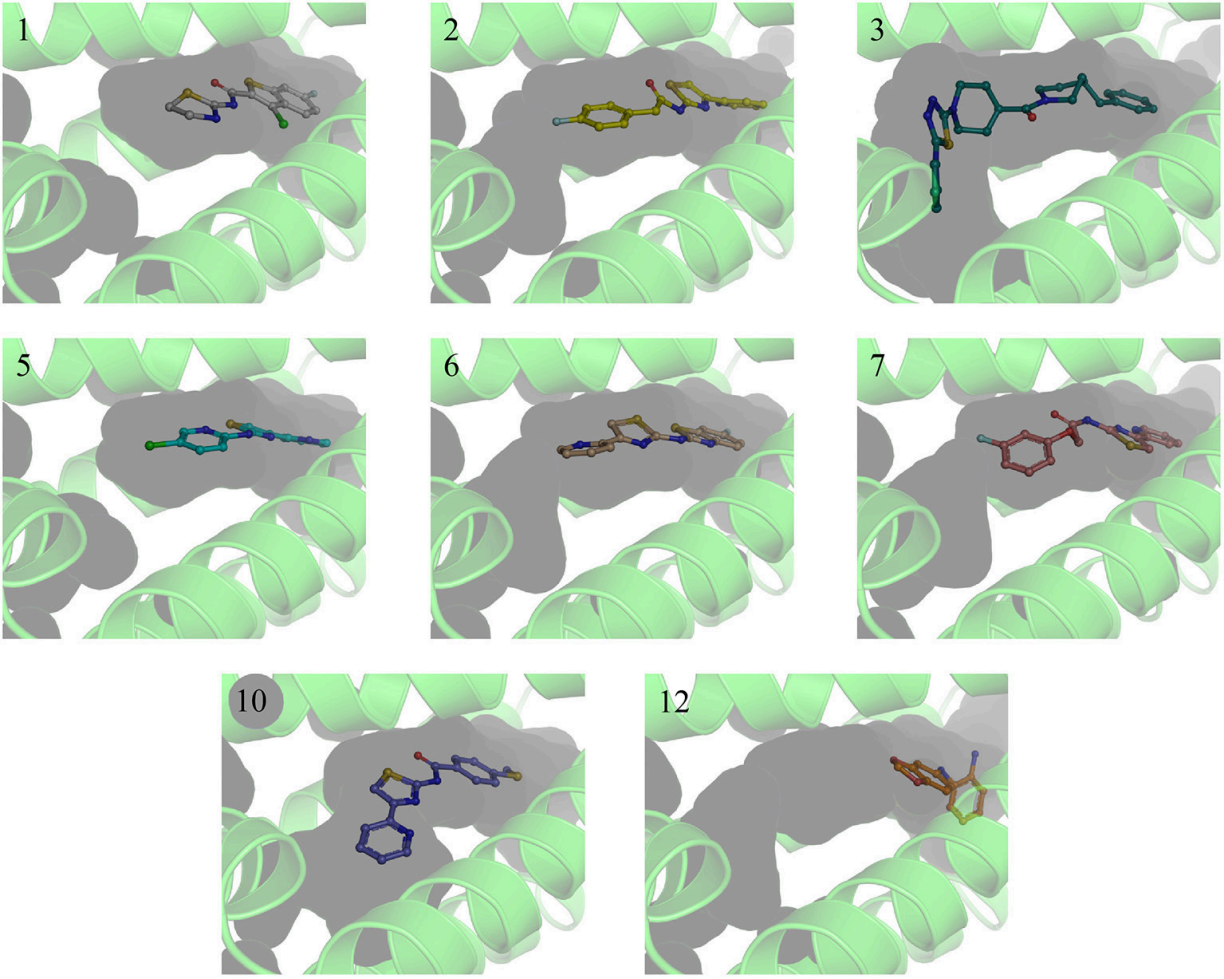
X-ray crystal structures of EthR:inhibitor complexes showing binding cavity surface and binding modes of the ligands. Numbers reflect the respective compounds. Figures were made using Pymol.

**Figure 4:**
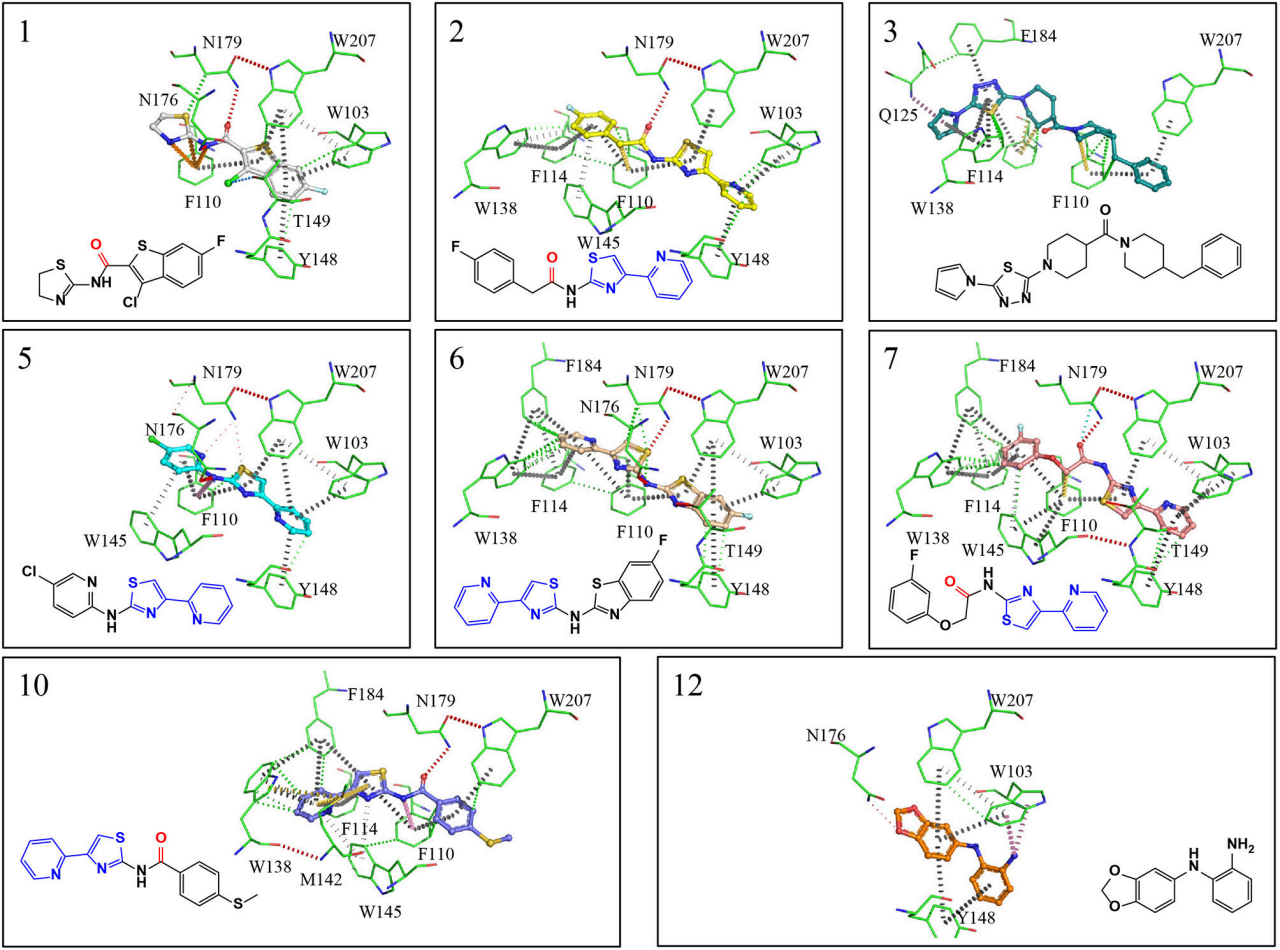
X-ray crystal structures showing interactions maps between EthR and inhibitors. Red disks represent hydrogen bonds, blue dots halogen bonds, gray disks depict π-π interactions, green disks hydrophobic interactions, yellow disks suphur-π and carbon-π interactions, pink disks donor-π interactions, orange disks cation-π interactions and cyan dots carbonyl interactions. Interactions were calculated using Intermezzo plugin for Pymol (Ochoa et al. unpublished). The carbonyl groups highlighted in red form hydrogen bonds with asn176 and are a common feature in many published EthR inhibitors. The common substructure (4-(pyridine-2-yl)thiazole) is highlighted in blue.

A single compound concentration run at 100 μM was performed using SPR to evaluate the inhibitory effect of individual compounds in EthR binding to DNA (Table S2A). From this run we could immediately observe that the 71% (25/35) of the compounds were active against EthR (> 50% inhibition) and that compounds that were outside the selection criteria all had low activities (inhibition < 60%), (Figures 1C,D), confirming that these computational methods can distinguish true and false negatives. The top 20 compounds were then selected to determine IC_50_ values (Table 2 and Figure 2). Amongst these were compounds **1**, **5**, **8**, **10**, **12**, and others identified solely from docking calculations, illustrating the complementarity of ligand-based and structure-based methods (Figures 1C,D). Eight of the tested compounds had an IC_50_ lower than 50 μM while for the others, accurate IC_50_ values were not possible to obtain due to solubility issues in the tested assay conditions. We then managed to obtain X-ray crystal structures for compounds **1**, **2**, **3**, **5**, **6**, **7**, **10**, and **12** bound to EthR (Figures 3, 4). Omit maps for each of the ligands is available in Figure S1.

**Table 2:** Inhibition, IC_50_ values, MIC, ligand efficiency (LE), and PDB codes for inhibitors **1–7, 10, 12, and 19**.

**Compound**	**Inhibition (100 μM) (%)**	**IC_50_**	**IC_50_ 95% confidence interval**	**MIC^#^ (μM)**	**LE**	**Structure**
**1** GSK1107112a	100	12	11–13	8.6	0.35	PDB code 5MXV
**2** GSK1570606a	100	9.9	8.2–12	9.3	0.31	PDB code 5MYL
**3** GSK2032710a	100	3.9	3.3–4.6	4.2	0.24	PDB code 5MYM
**4** GSK735826a	100	13	11–15	2.7	0.28	Not obtained
**5** GSK445886a	99	30	22–41	5.9	0.34	PDB code 5MYN
**6** GSK735816a	97	22	20–25	2.3	0.30	PDB code 5MYR
**7** GSK920684a	93	50	25–101	3.5	0.26	PDB code 5MYS
**10** GSK921295a	74	^*^	^*^	8.7		PDB code 5MYT
**12** SB-435634	67	^*^	^*^	1.8		PDB code 5MYW
**19** GSK2157753a	58	45	42–48	8.3	0.28	Not obtained

* *IC_50_ for these compounds was not possible to obtain in the tested conditions due to solubility issues. #MIC data was published before (Ballell et al., 2013)*.

EthR binding site is characterized by a long hydrophobic tunnel surrounded mostly by hydrophobic residues, many of which are aromatic, plus four polar amino acids and a basic residue (Dover et al., 2004). The interactions of our compounds with EthR were dominated π–π interactions with (W103, F110, F114, W138, W145, Y148, F184, and W207, although hydrogen bonds were also present, with all but compound **3** forming at least one hydrogen bond with one of the polar residues (T149, N176, and N179) as seen in Figure 4. Other interactions such as hydrogen donor-π or cation-π (observed in compound **1** (F110), **3** (Q125), **5** (F110), **10** (F110), and **12** (W103), sulfur-π (observed in compound **10** with M142), carbon-π (observed in compound **2**, **3** and **7**), and halogen bond (observed in compound **1** with T149) were also present but less prevalent (Figure 4). Hydrophobic interactions, extending from W103 at the entrance of the binding site to W138 at the inner hydrophobic site are also observed for many of the compounds (Figure 4). This dense network of protein-ligand interactions appears to stabilize the conformational changes in EthR and inhibition of DNA binding observed by SPR. Interestingly six of the best compounds (**2, 4, 5, 6, 7, 10**) share a common substructure composed by a 4-(pyridine-2-yl)thiazole (Figure 4), that for compounds **2, 5**, and **7** occupies the same part of EthR binding site forming π-interactions with F110, W103, Y148, and W207, while for compounds **6** and **10** the same substructure forms π-interactions with F110, F114, W138, W145, and F184 (Figure 4). Another common feature shared between several of the compounds (**1, 2, 3, 7**, and **10**) is the presence of a ketone that forms a hydrogen bond with N176 in all but compound **3**. This ketone is a feature shared by many other EthR binding compounds reported in the literature (Figure 4 and Figure S2). Although we did not obtain a structure for one of the most potent EthR inhibitors identified in this work (compound **4**, IC_50_ ≈ 13 μM) (Table 2), it is quite likely that this compound, like others reported here, interacts with EthR by forming similar interactions at the binding site.

### Validation of InhA inhibitors

**Figure 5:**
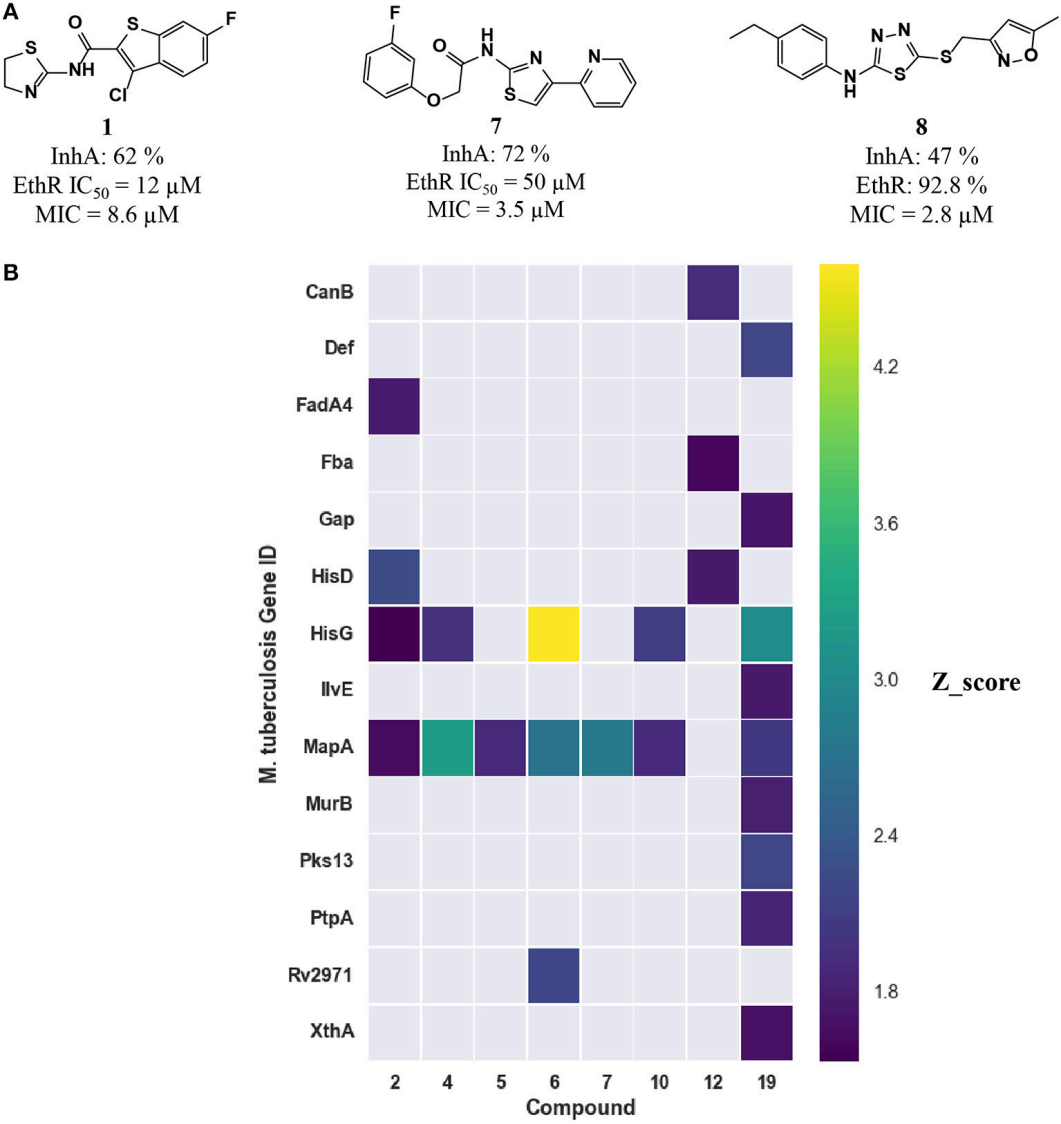
**(A)** Three inhibitors exhibited dual inhibitory activity against EthR and InhA. **(B)** Heatmap displaying the other *M. tuberculosis* essential proteins predicted using MCNBC for the top 8 EthR inhibitors.

To assess whether the predicted compounds are active against InhA, an enzymatic assay was performed with compounds tested at a single concentration of 100 μM. Only compounds **1**, **7**, and **8** showed inhibition greater than 40% (Figure 5A). IC_50_s were not possible to calculate due to solubility issues in the required assay conditions. An attempt to address this issue by increasing DMSO concentration, to improve compound solubility, led to very low enzymatic activity that prevented obtaining reliable data.

### Dual activity against EthR and InhA

The computational target prediction approaches described here have highlighted the possibility of inhibiting two or more *M. tuberculosis* proteins by one of the phenotypic hits. Amongst the 35 compounds selected for *in vitro* assays against EthR, 17 compounds were also predicted to hit InhA. Three compounds displayed inhibitory activity against both EthR and InhA (Figure 5A). Compound **1** is active against both EthR (IC_50_ ≈12 μM) and InhA (62% inhibition at 100 μM) (Table 2), confirming the computational predictions. Interestingly, this compound was identified solely from structure-based predictions. Even though an attempt to obtain a co-crystal structure of the compound with InhA was unsuccessful, the docking LEI values for the compound to both enzymes (EthR: 1.58 and InhA: 1.11) indicate high binding affinity. The EthR-compound **1** co-crystal structure confirms the occupancy of the compound in the EthR binding site.

Although targeting EthR has been reported to boost the activity of the second-line drug ethionamide against InhA (Willand et al., 2009), EthR itself is not an essential protein and compounds that solely hit EthR will not show sterilizing activity without the concomitant use of ethionamide. Furthermore, the dual activity compounds reported here, are not sufficiently potent *in vitro* inhibitors of InhA to explain their observed MIC value. This indicates that although many of our predictions were confirmed, the reasons behind whole cell activities of the compounds most likely lie elsewhere.

Interestingly some of the compounds reported here have chemical groups that are known to be modified by EthA. Thiophenes and thioethers are present in compounds **1, 8, and 10** and are known to be substrates for EthA (Mori et al., 2015; Grant et al., 2016). Furthermore, EthA was found to have a much wider substrate preference than initially thought and is known to modify also thiocarbamides and thioureas (Dover et al., 2007; Nishida and Ortiz de Montellano, 2011; Gopal and Dick, 2015; Grzegorzewicz et al., 2015). Although speculative it is possible that these compounds hit EthR but are also pro-drugs that are activated by EthA self-increasing their own activation by raising the levels of EthA available inside *M. tuberculosis* and subsequently hitting other targets.

A more conservative explanation is that the observed MICs are explained by a direct dual or polypharmacology by hitting other *M. tuberculosis* targets. To that effect, the heatmap in Figure 4 displays the 16 essential *M. tuberculosis* proteins predicted for eight EthR inhibitors (compounds **2**, **4**, **5**, **6**, **7**, **10**, **12**, and **19**) using ligand-based approach. The other three inhibitors, **1**, **3**, and **8** were identified through a structure-based approach, hence are not shown in Figure 4. We observe that each compound has the potential to hit at least one essential protein, with MapA assigned for all but compound **12** (Figure 5B) and compound **19** potentially targeting 10 proteins. Furthermore, compounds **5** and **12** selected for both EthR and InhA from the structure-based predictions were assigned to further essential *M. tuberculosis* proteins by the ligand-based MCNBC method. A summary of the all the other predicted *M. tuberculosis* proteins for these compounds is given in Table S4. However, it is also possible that the observed MIC values are a result of a synergistic effect that also includes their activities against InhA and EthR and on other enzyme(s).

A designed multi-targeted approach in drug discovery has been attracting attention in recent years, with large scale functional genomics studies in several model organisms. Many single gene knockouts by themselves show no, or small, phenotypic effect with as little as ~19% of the genes being essential in a number of model organisms (Hopkins, 2008). Compounds that hit multiple targets have the capacity to explore synthetic lethal interaction that further expand the number of available targets in an organism (Hopkins, 2008). The fact that many of these compounds show strong EthR inhibition together with very good MIC values raises the prospect of further enhancing ethionamide killing effect with a combined therapy that uses an ethionamide boosting compound that also kills *M. tuberculosis* by hitting one or more enzymes.

Several other TB drugs thiacetazone, thiocarlide (isoxyl), and the recently developed perchlozone have been shown to be activated by EthA and hit the same target, HadABC, a group of enzymes involved in mycolic acid synthesis (Gopal and Dick, 2015; Grzegorzewicz et al., 2015). Thiacetazone use has declined due to severe and sometimes fatal side effects (Miller et al., 1972), and isoxyl is no longer used because of failures in clinical outcomes due to its low plasma concentrations (Wang et al., 2012). Potentiating the activation of these drugs by increasing the amount of EthA available in the cells with EthR inhibitors might allow some of these drugs to be viable in modern TB chemotherapy by lowering the dosage required to kill *M. tuberculosis*. Perchlozone is a new TB drug approved in the Russian Federation to treat MDR-TB. Again, EthR inhibitors may potentiate the killing effects of this drug by increasing the amount of EthA available making it a much stronger anti-TB drug.

## Author contributions

GM and GP generated the multi-category naive Bayesian models. GM designed the chemogenomic target prediction protocol, performed all ligand-based and structure-based target prediction experiments, analyzed the results, and selected target-compounds pairs for experimental validation. JL, LB, and DB prepared and provided all samples of the *M. tuberculosis* phenotypic hits. VM and MB cloned and purified the proteins, designed, and performed all biophysical and structural experiments. MS performed InhA enzymatic assays and synthesized InhA substrate. JPO, TLB, and CA supervised the work. GM and VM wrote the manuscript. All authors revised the manuscript.

### Conflict of interest statement

The authors declare that the research was conducted in the absence of any commercial or financial relationships that could be construed as a potential conflict of interest. The reviewer AM and handling Editor declared their shared affiliation.
