# Supplementary material for: Target Identification of Mycobacterium tuberculosis Phenotypic Hits Using a Concerted Chemogenomic, Biophysical, and Structural Approach
Source: Front Pharmacol. 2017 Sep 26;8:681. doi: 10.3389/fphar.2017.00681 (PMC5623190; doi:10.3389/fphar.2017.00681)
Supplement: Supplementary file 1 [file DataSheet1.DOCX]

**Supplementary Information**

**Target identification of *Mycobacterium tuberculosis* phenotypic hits using a concerted chemogenomic, biophysical and structural approach**

Grace Mugumbate^1,2*^#, Vitor Mendes^2*^#, Michal Blaszczyk^2^#, Mohamad Sabbah^3^, George Papadatos^1^§, Joel Lelievre^4^, Lluis Ballell^4^, David Barros^4^, Chris Abell^3^, Tom L. Blundell^2^ and John P. Overington^1,5^

1 - European Molecular Biology Laboratory – European Bioinformatics Institute (EMBL-EBI), Wellcome Trust Genome Campus, Hinxton, CB10 1SD, United Kingdom

2 - Department of Biochemistry, University of Cambridge, Tennis Court Road, Cambridge CB1 2GA, United Kingdom.

3 - Department of Chemistry, University of Cambridge, Lensfield Road, Cambridge CB2 1EW, United Kingdom.

4 - Diseases of the Developing World, GlaxoSmithKline, Severo Ochoa 2, 28760 Tres Cantos, Madrid, Spain.

5 - Medicines Discovery Catapult, Mereside, Alderley Park, Alderley Edge, Cheshire, United Kingdom.

§ - Current address: GlaxoSmithKline, Medicines Research Centre, Gunnels Wood Road, Stevenage, Herts SG1 2NY, UK.

^*^ Corresponding authors:

[grace@ebi.ac.uk](mailto:grace@ebi.ac.uk)

[vgm23@cam.ac.uk](mailto:vgm23@cam.ac.uk)

# These authors contributed equally to this work and are co-first authors.

**Supplementary Methods**

**Table S1:** Crystallographic and data collection statistics

| Compound | **1** | **2** | **3** | **5** | **6** | **7** | **10** | **12** |
| --- | --- | --- | --- | --- | --- | --- | --- | --- |
| PDB ID | 5MXV | 5MYL | 5MYM | 5MYN | 5MYR | 5MYS | 5MYT | 5MYW |
| **Data collection*** |  |  |  |  |  |  |  |  |
| Space group | *P*4_1_2_1_2 | *P*4_1_2_1_2 | *P*2 | *P*4_1_2_1_2 | *P*4_1_2_1_2 | *P*4_1_2_1_2 | *P*4_1_2_1_2 | *P*4_1_2_1_2 |
| Cell parameters:  a [Å]  b [Å]  c [Å]  α=β=γ=90˚ (or β for *P*2) | 121.23  121.23  33.84 | 120.66  120.66  33.66 | 86.62  56.48  98.19  90.01 | 121.81  121.81  33.70 | 121.36  121.36  33.70 | 121.55  121.55  33.79 | 121.17  121.17  33.72 | 121.53  121.53  33.76 |
| Resolution range [Å] | 121.23 – 1.62  (1.71 – 1.62) | 42.66 – 1.72  (1.82 – 1.72) | 98.19 – 2.28  (2.40 – 2.28) | 43.07 – 1.56  (1.65 – 1.56) | 121.36 – 1.83  (1.93 – 1.83) | 121.55 – 1.59  (1.67 – 1.59) | 38.32 – 1.61  (1.70 – 1.61) | 60.76 – 1.77  (1.86 – 1.77) |
| No. of observations  total  unique | 713630  (54085)  32415  (4638) | 331256  (44027)  26955  (3849) | 146825  (21103)  43150  (6247) | 396526  (26057)  36051  (4725) | 290306  (42469)  22975  (3275) | 431353  (51807)  34673  (4822) | 375730  (30076)  32287  (3884) | 316819  (46830)  25356  (3617) |
| R_merge_ | 0.096(1.035) | 0.077 (1.277) | 0.058 (0.461) | 0.054 (0.767) | 0.107 (1.439) | 0.061 (1.141) | 0.089 (1.494) | 0.077 (1.340) |
| I/σ(I) | 26.6 (2.3) | 18.7 (1.9) | 12.1 (2.2) | 26.0 (1.9) | 17.4 (2.3) | 22.0 (2.1) | 20.3 (1.9) | 19.1 (2.3) |
| Completeness [%] | 100.0 (99.9) | 100.0 (100.0) | 98.7 (98.5) | 98.8 (92.4) | 100.0 (99.8) | 99.2 (96.5) | 97.5 (84.1) | 99.7 (99.5) |
| Multiplicity | 22.0 (11.7) | 12.3 (11.4) | 3.4 (3.4) | 11.0 (5.5) | 12.6 (13.0) | 12.4 (10.7) | 11.6 (7.7) | 12.5 (12.9) |
| **Refinement** |  |  |  |  |  |  |  |  |
| Refinement program | PHENIX | PHENIX | PHENIX | PHENIX | PHENIX | PHENIX | PHENIX | PHENIX |
| Resolution [Å] | 85.72 – 1.63 | 28.56 – 1.72 | 98.19 – 2.28 | 43.07 – 1.56 | 85.82 – 1.83 | 85.95 – 1.59 | 38.32 – 1.61 | 33.71 – 1.77 |
| No. reflections | 32356 | 26884 | 43140 | 35991 | 22915 | 34616 | 32185 | 25300 |
| R_work_/R_free_ [%] | 17.1/19.2 | 18.4/21.5 | 17.8/22.7 | 17.1/19.5 | 17.7/20.3 | 17.5/20.8 | 16.9/17.8 | 17.7/19.8 |
| RMS deviations |  |  |  |  |  |  |  |  |
| Bonds [Å] | 0.006 | 0.006 | 0.008 | 0.006 | 0.007 | 0.006 | 0.005 | 0.006 |
| Angles [˚] | 0.783 | 0.735 | 0.985 | 0.713 | 0.885 | 0.772 | 0.752 | 0.761 |
| Ramachandran |  |  |  |  |  |  |  |  |
| Favoured [%] | 99 | 99 | 99 | 99 | 99 | 99 | 98 | 99 |
| Outliers [%] | 0 | 0 | 0 | 0 | 0 | 0 | 0 | 0 |
| Mean ADP  Protein [Å^2^]  Ligand [Å^2^] | 39.4  40.0 | 43.7  72.0 | 46.2  70.0 | 35.7  37.3 | 38.8  72.5 | 37.5  75.0 | 38.0  44.3 | 47.2  67.0 |

* Parameters shown in brackets are for the highest resolution shell

**Supplementary Results**

**Table S2a:** Number of compounds for each predicted *M. tuberculosis* target using MCNBC. Targets are ranked in descending order of number of assigned ligands

|  | *M. tuberculosis Gene id* | Essentiality* | *M. tuberculosis* UniProt Accession | *M. tuberculosis* Protein Name | Number of compounds |
| --- | --- | --- | --- | --- | --- |
| 1 | *Rv2234*  *ptpA* | Y([1](#_ENREF_1)) | P65716 | Probable low molecular weight protein-tyrosine-phosphatase (PTPase) (EC 3.1.3.48) | 102 |
| 2 | *Rv1527c*  *pks5* | N([2](#_ENREF_2)) | O53901 | Probable polyketide synthase pks5 | 100 |
| 3 | *Rv2933*  *ppsC* | N([2](#_ENREF_2)) | P96202 | Phthiocerol synthesis polyketide synthase type I PpsC (Beta-ketoacyl-acyl-carrier-protein synthase I) (EC 2.3.1.41) | 100 |
| 4 | *Rv0363c*  *fba* | Y([2](#_ENREF_2)) | P67475 | Fructose-bisphosphate aldolase (FBP aldolase) (FBPA) (EC 4.1.2.13) (Fructose-1,6-bisphosphate aldolase) | 99 |
| 5 | *Rv2935*  *ppsE* | N([2](#_ENREF_2)) | P96204 | Phthiocerol synthesis polyketide synthase type I PpsE (Beta-ketoacyl-acyl-carrier-protein synthase I) (EC 2.3.1.41) | 98 |
| 6 | Rv1181  *pks4* | N([2](#_ENREF_2)) | O50437 | Probale polyketide beta-ketoacyl synthase Pks4 EC 2.3.1.-) | 97 |
| 7 | *Rv2934*  *ppsD* | N([2](#_ENREF_2)) | P96203 | Phthiocerol synthesis polyketide synthase type I PpsD (Beta-ketoacyl-acyl-carrier-protein synthase I) (EC 2.3.1.41) | 95 |
| 8 | *Rv2121c*  *hisG* | Y([2](#_ENREF_2)) | P60759 | ATP phosphoribosyltransferase (ATP-PRT) (ATP-PRTase) (EC 2.4.2.17) | 94 |
| 9 | *Rv1661*  *pks7* | N([2](#_ENREF_2)) | P94996 | Polyketide synthase (Probable polyketide synthase pks7) | 94 |
| 10 | *Rv0734*  *mapA* | Y([3](#_ENREF_3)) | Q7D9D5 | Methionine aminopeptidase 1 (MAP) (EC 3.4.11.18) (Peptidase M) | 94 |
| 11 | *Rv2048c*  *pks12* | Y([4](#_ENREF_4)) | O53490 | Probable polyketide synthase pks12 | 92 |
| 12 | *Rv3800c*  *pks13* | Y([2](#_ENREF_2)) | O53579 | Polyketide synthase Pks13 (Polyketide synthase) | 92 |
| 13 | *Rv1662*  *pks8* | N([2](#_ENREF_2)) | O65933 | Probable polyketide synthase pks8 | 91 |
| 14 | *Rv2931*  *ppsA* | N([2](#_ENREF_2)) | Q10977 | Phthiocerol synthesis polyketide synthase type I PpsA (Beta-ketoacyl-acyl-carrier-protein synthase I) (EC 2.3.1.41) | 91 |
| 15 | *Rv1664*  *pks9* | N([2](#_ENREF_2)) | O06586 | Polyketide synthase (Probable polyketide synthase pks9) | 91 |
| 16 | *Rv0405*  *pks6* | N([5](#_ENREF_5)) | O86335 | Probable membrane bound polyketide synthase polyketide synthase Pks6 | 90 |
| 17 | *Rv3855*  *ethR* | N([2](#_ENREF_2)) | P96222 | HTH-type transcriptional regulator EthR | 89 |
| 18 | *Rv1180*  *pks3* | N([2](#_ENREF_2)) | O50436 | Probable polyketide beta-ketoacyl synthase Pks3 (EC 2.3.1.-) | 87 |
| 19 | *Rv2940c*  *mas* | N([2](#_ENREF_2)) | P96291 | Mycocerosic acid synthase (probable multifunctional mycocerosic acid synthase membrane-associated Mas) | 87 |
| 20 | *Rv2932*  *ppsB* | N([2](#_ENREF_2)) | Q10978 | Phthiocerol synthesis polyketide synthase type I PpsB (Beta-ketoacyl-acyl-carrier-protein synthase I) (EC 2.3.1.41) | 87 |
| 21 | *Rv2068c*  *blaC* | N([2](#_ENREF_2)) | P0C5C1 | Beta-lactamase (EC 3.5.2.6) (Penicillinase) | 87 |
| 22 | *Rv2946c*  *pks1* | N([2](#_ENREF_2)) | P96285 | Putative inactive phenolphthiocerol synthesis polyketide synthase type I Pks1 | 81 |
| 23 | *Rv2781c* | N([4](#_ENREF_4)) | O33323 | 2-nitropropane dioxygenase, putative (possible alanine rich oxireductase) (EC 1.-.-.-) | 79 |
| 24 | *Rv2947c*  *pks15* | N([2](#_ENREF_2)) | P96284 | Putative inactive phenolphthiocerol synthesis polyketide synthase type I Pks15 | 75 |
| 25 | *Rv2861c*  *mapB* | N([3](#_ENREF_3)) | P0A5J2 | Methionine aminopeptidase 2 (MAP) (EC 3.4.11.18) (Peptidase M) | 74 |
| 26 | *Rv0435c* | N([2](#_ENREF_2)) | P96281 | Cell division control protein, putative (putative conserved ATPase) (EC 3.6.1.-) | 71 |
| 27 | *Rv3553* | N([2](#_ENREF_2)) | P71847 | Possible oxireductase (EC 1.-.-.-) | 69 |
| 28 | *Rv0533c*  *fabH* | N([2](#_ENREF_2)) | P0A574 | 3-oxoacyl-[acyl-carrier-protein] synthase 3 (EC 2.3.1.180) (3-oxoacyl-[acyl-carrier-protein] synthase III) (Beta-ketoacyl-ACP synthase III) (KAS III) (MtFabH) | 67 |
| 29 | *Rv3197* | N([2](#_ENREF_2)) | O53343 | Probable conserved ATP-Binding Protein ABC transporter | 65 |
| 30 | *Rv2045c*  *lipT* | N([2](#_ENREF_2)) | O53488 | Probable carboxylesterase LipT (EC 3.1.1.-) | 63 |
| 31 | *Rv3588c*  *canB* | Y([4](#_ENREF_4)) | O53573 | Carbonic anhydrase (EC 4.2.1.1) (Carbonate dehydratase) | 60 |
| 32 | *Rv2210c*  *ilvE* | Y([2](#_ENREF_2)) | Q10399 | Branched-chain-amino-acid aminotransferase (BCAT) (EC 2.6.1.42) | 57 |
| 33 | *Rv1484*  *inhA* | Y([2](#_ENREF_2)) | P0A5Y6 | Enoyl-[acyl-carrier-protein] reductase [NADH] (EC 1.3.1.9) (NADH-dependent enoyl-ACP reductase) | 57 |
| 34 | *Rv3825c*  *pks2* | N([2](#_ENREF_2)) | O07798 | Phthioceranic/hydroxyphthioceranic acid synthase (EC 2.3.1.-) (Polyketide synthase pks2) | 55 |
| 35 | *Rv1640c*  *lysX* | Y([6](#_ENREF_6)) | P94974 | Lysylphosphatidylglycerol biosynthesis bifunctional protein LysX [Includes: Lysine--tRNA ligase (EC 6.1.1.6) (Lysyl-tRNA synthetase) (LysRS); Phosphatidylglycerol lysyltransferase (EC 2.3.2.3) (Lysylphosphatidylglycerol synthetase) (LPG synthetase)] | 54 |
| 36 | *Rv3598c*  *lysS* | Y([2](#_ENREF_2)) | P67607 | Lysine--tRNA ligase 1 (EC 6.1.1.6) (Lysyl-tRNA synthetase 1) (LysRS 1) | 52 |
| 37 | *Rv0902c*  *prrB* | Y([7](#_ENREF_7)) | P0A5Z8 | Sensor-type histidine kinase prrB (EC 2.7.13.3) | 51 |
| 38 | *Rv2737c*  *recA* | N([8](#_ENREF_8)) | P0A5U4 | Protein RecA (Recombinase A) [Cleaved into: Endonuclease PI-MtuI (EC 3.1.-.-) (Mtu RecA intein)] | 51 |
| 39 | *Rv0427c*  *xthA* | Y([4](#_ENREF_4)) | P96273 | Exodeoxyribonuclease III (EC 3.1.11.2) | 43 |
| 40 | *Rv1938*  *ephB* | N([2](#_ENREF_2)) | P95276 | Epoxide hydrolase (Probable Epoxide Hydrolase EphB (Epoxide Hydratase)) (EC 3.3.2.3) | 42 |
| 41 | *Rv2361c*  *uppS* | Y([2](#_ENREF_2)) | P60479 | Undecaprenyl pyrophosphate synthase (UPP synthase) (EC 2.5.1.31) (Di-trans,poly-cis-decaprenylcistransferase) (Ditrans,polycis-undecaprenyl-diphosphate synthase) (Undecaprenyl diphosphate synthase) (UDS) | 40 |
| 42 | *Rv0429c*  *Def* | Y([2](#_ENREF_2)) | P96275 | Peptide deformylase (PDF) (EC 3.5.1.88) (Polypeptide deformylase) | 40 |
| 43 | *Rv3608c*  *folP1* | Y([2](#_ENREF_2)) | P0A578 | Dihydropteroate synthase 1 (DHPS 1) (EC 2.5.1.15) (Dihydropteroate pyrophosphorylase 1) | 37 |
| 44 | *Rv2523c*  *acpS* | N([2](#_ENREF_2)) | P0A4W8 | Holo-[acyl-carrier-protein] synthase (Holo-ACP synthase) (EC 2.7.8.7) (4'-phosphopantetheinyl transferase AcpS) | 37 |
| 45 | *Rv2443*  *dctA* | N([2](#_ENREF_2)) | P71906 | C4-dicarboxylate transport protein (probable C4-dicarboxylate-transport transmembrane protein Dcta) | 37 |
| 46 | *Rv1730c* | N([2](#_ENREF_2)) | P71988 | Possible penicillin-binding protein | 36 |
| 47 | *Rv2870c*  *Dxr* | Y([9](#_ENREF_9)) | P64012 | 1-deoxy-D-xylulose 5-phosphate reductoisomerase (DXP reductoisomerase) (EC 1.1.1.267) | 36 |
| 48 | *Rv1436*  *gap* | Y([2](#_ENREF_2)) | P64178 | Glyceraldehyde-3-phosphate dehydrogenase (GAPDH) (EC 1.2.1.12) | 34 |
| 49 | *Rv0482*  *murB* | Y([2](#_ENREF_2)) | P65460 | UDP-N-acetylenolpyruvoylglucosamine reductase (EC 1.1.1.158) (UDP-N-acetylmuramate dehydrogenase) | 33 |
| 50 | *Rv3670*  *ephE* | N([2](#_ENREF_2)) | O69638 | Hydrolase, alpha/beta hydrolase fold family (possible epoxide hydrolase EphE (epoxide hydratase) (arene-oxide hydratase)) (EC 3.3.2.3) | 31 |
| 51 | *Rv3273* | N([2](#_ENREF_2)) | P96878 | Probable transmembrane carbonic anhydrase (carbonate dehydratase) (carbonic dehydratase) (EC 4.2.1.1) (Sulfate transporter/carbonic anhydrase, putative) | 30 |
| 52 | *Rv2537c*  *aroD* | Y([2](#_ENREF_2)) | P0A4Z6 | 3-dehydroquinate dehydratase (3-dehydroquinase) (EC 4.2.1.10) (Type II DHQase) | 30 |
| 53 | *Rv1284*  *canA* | Y([4](#_ENREF_4)) | P64797 | Putative carbonate dehydratase-like protein Rv1284 (EC 4.2.1.-) | 30 |
| 54 | *Rv1599*  *hisD* | Y([2](#_ENREF_2)) | P63950 | Histidinol dehydrogenase (HDH) (EC 1.1.1.23) | 29 |
| 55 | *Rv0764c*  *cyp51* | N([2](#_ENREF_2)) | P0A512 | Lanosterol 14-alpha demethylase (EC 1.14.13.70) (CYPLI) (Cytochrome P450 51) (Cytochrome P450-14DM) (Cytochrome P450-LIA1) (Sterol 14-alpha demethylase) | 29 |
| 56 | *Rv3627c* | Y([2](#_ENREF_2)) | O06380 | Putative uncharacterized protein | 29 |
| 57 | *Rv2971* | Y([2](#_ENREF_2)) | P95124 | Uncharacterized oxidoreductase Rv2971/MT3049 (EC 1.-.-.-) | 29 |
| 58 | *Rv1707* | N([2](#_ENREF_2)) | O33206 | Probable conserved transmembrane protein (Sulfate transporter) | 28 |
| 59 | *Rv1086* | N([2](#_ENREF_2)) | O53434 | Short-chain Z-isoprenyl diphosphate synthase (EC 2.5.1.68) ((2Z,6E)-farnesyl diphosphate synthase) (Z-FPP synthase) (Z-FPPS) (Z-isoprenyl diphosphate synthase) | 28 |
| 60 | *Rv3170*  *aofH* | N([2](#_ENREF_2)) | P63533 | Putative flavin-containing monoamine oxidase AofH (EC 1.4.3.-) | 27 |
| 61 | *Rv3062*  *ligB* | N([2](#_ENREF_2)) | P95096 | Probable DNA ligase (EC 6.5.1.1) (Polydeoxyribonucleotide synthase [ATP]) | 27 |
| 62 | *Rv2764c*  *thyA* | N([10](#_ENREF_10)) | P67044 | Thymidylate synthase (TS) (TSase) (EC 2.1.1.45) | 26 |
| 63 | *Rv0153c*  *ptbB* | N([2](#_ENREF_2)) | P96830 | Phosphotyrosine protein phosphatase PtpB (protein-tyrosine-phosphatase) (PTPase) (EC 3.1.3.48) | 26 |
| 64 | *Rv0373c* | N([2](#_ENREF_2)) | O53708 | Carbon monoxide dehydrogenase, large subunit, putative (probable carbon monoxide dehydrogenase (large chain)) (EC 1.2.99.2) | 26 |
| 65 | *Rv1323*  *fadA4* | Y([4](#_ENREF_4)) | P66926 | Probable acetyl-CoA acetyltransferase (EC 2.3.1.9) (Acetoacetyl-CoA thiolase) | 25 |
| 66 | *Rv2763c*  *dfrA* | Y([2](#_ENREF_2)) | P0A546 | Dihydrofolate reductase (EC 1.5.1.3) | 25 |
| 67 | *Rv0091*  *Mtn* | N([2](#_ENREF_2)) | P67656 | MTA/SAH nucleosidase (EC 3.2.2.9) (5'-methylthioadenosine nucleosidase) (S-adenosylhomocysteine nucleosidase) | 23 |
| 68 | *Rv1338*  *murI* | Y([11](#_ENREF_11)) | P63635 | Glutamate racemase (EC 5.1.1.3) | 22 |
| 69 | *Rv1747* | Y([12](#_ENREF_12)) | O65934 | Probable conserved transmembrane ATP-binding protein ABC transporter | 22 |
| 70 | *Rv2110c*  *prcB* | Y([13](#_ENREF_13)) | O33245 | Proteasome subunit beta (EC 3.4.25.1) (20S proteasome beta subunit) (Proteasome core protein PrcB) | 19 |
| 71 | *Rv1536*  *ileS* | Y([2](#_ENREF_2)) | Q10765 | Isoleucine--tRNA ligase (EC 6.1.1.5) (Isoleucyl-tRNA synthetase) (IleRS) | 18 |
| 72 | *Rv2299c*  *htpG* | N([2](#_ENREF_2)) | P64411 | Chaperone protein htpG (Heat shock protein htpG) (High temperature protein G) | 18 |
| 73 | *Rv2182c* | Y([2](#_ENREF_2)) | O53516 | 1-acylglycerol-3-phosphate O-acyltransferase (EC 2.3.1.51) (Acyltransferase family protein) | 18 |
| 74 | *Rv2139*  *pyrD* | Y([2](#_ENREF_2)) | P65908 | Dihydroorotate dehydrogenase (quinone) (EC 1.3.5.2) (DHOdehase) (DHOD) (DHODase) (Dihydroorotate oxidase) | 18 |
| 75 | *Rv3617*  *ephA* | N([5](#_ENREF_5)) | O06266 | Epoxide hydrolase (EC 3.3.2.9) (probable epoxide hydrolase EphA (epoxide hydratase) (arene-oxide hydratase)) (EC 3.3.2.3) | 17 |
| 76 | *Rv1981c*  *nrdF1* | N([2](#_ENREF_2)) | Q10840 | Ribonucleoside-diphosphate reductase subunit beta nrdF1 (EC 1.17.4.1) (Ribonucleotide reductase R2-1 small subunit) (Ribonucleotide reductase small subunit 1) | 16 |
| 77 | *Rv2855*  *Mtr* | N([2](#_ENREF_2)) | O07927 | Mycothione reductase (EC 1.8.1.15) (Mycothiol-disulfide reductase) (NADPH-dependent mycothione reductase) | 16 |
| 78 | *Rv1649*  *pheS* | Y([2](#_ENREF_2)) | P94984 | Phenylalanine--tRNA ligase alpha subunit (EC 6.1.1.20) (Phenylalanyl-tRNA synthetase alpha subunit) (PheRS) | 16 |
| 79 | *Rv2483c*  *plsC* | Y([4](#_ENREF_4)) | O53208 | Acyltransferase family protein | 16 |
| 80 | *Rv3218* | N([2](#_ENREF_2)) | O05848 | Diacylglycerol kinase catalytic domain-containing protein | 15 |
| 81 | *Rv3602c*  *panC* | N([14](#_ENREF_14)) | P0A5R0 | Pantothenate synthetase (PS) (EC 6.3.2.1) (Pantoate--beta-alanine ligase) (Pantoate-activating enzyme) | 15 |
| 82 | *Rv2677c*  *hemY* | Y([2](#_ENREF_2)) | P0A5A7 | Protoporphyrinogen oxidase (PPO) (EC 1.3.3.4) | 15 |
| 83 | *Rv0183* | N([2](#_ENREF_2)) | O07427 | Possible lysophospholipase (EC 3.1.-.-) | 15 |
| 84 | *Rv2384*  *mbtA* | N([2](#_ENREF_2)) | P71716 | 2,3-dihydroxybenzoate-AMP ligase (bifunctional enzyme Mbta: salicyl-AMP ligase (SAL-AMP ligase) + salicyl-S-ArCP synthetase) (EC 6.-.-.-) | 15 |
| 85 | *Rv1007c*  *metS* | Y([2](#_ENREF_2)) | O05593 | Methionine--tRNA ligase (EC 6.1.1.10) (Methionyl-tRNA synthetase) (MetRS) | 14 |
| 86 | *Rv3397c*  *phyA* | N([4](#_ENREF_4)) | P65860 | Probable phytoene synthase (EC 2.5.1.32) | 14 |
| 87 | *Rv0005*  *gyrB* | Y([2](#_ENREF_2)) | P0C5C5 | DNA gyrase subunit B (EC 5.99.1.3) | 14 |
| 88 | *Rv0233*  *nrdB* | N([2](#_ENREF_2)) | P96416 | R2-like ligand binding oxidase (EC 1.-.-.-) (Ribonucleotide reductase R2 subunit homolog) (Ribonucleotide reductase small subunit homolog) | 13 |
| 89 | *Rv0211*  *pckA* | N([2](#_ENREF_2)) | P65686 | Phosphoenolpyruvate carboxykinase [GTP] (PEP carboxykinase) (PEPCK) (EC 4.1.1.32) (Phosphoenolpyruvate carboxylase) | 13 |
| 90 | *Rv3255c*  *manA* | Y([2](#_ENREF_2)) | O05898 | Mannose-6-phosphate isomerase (EC 5.3.1.8) | 13 |
| 91 | *Rv1916*  *aceAb* | N([2](#_ENREF_2)) | O07717 | Probable isocitrate lyase aceAb [second part] (isocitrase) (isocitratase) (ICL) (EC 4.1.3.1) | 13 |
| 92 | *Rv1629*  *polA* | Y([2](#_ENREF_2)) | P0A550 | DNA polymerase I (POL I) (EC 2.7.7.7) | 12 |
| 93 | *Rv3314c*  *deoA* | N([2](#_ENREF_2)) | O53366 | Thymidine phosphorylase (EC 2.4.2.4) (TdRPase) | 11 |
| 94 | *Rv1617*  *pykA* | Y([2](#_ENREF_2)) | O06134 | Pyruvate kinase (PK) (EC 2.7.1.40) | 11 |
| 95 | *Rv2614c*  *thrS* | Y([2](#_ENREF_2)) | P67582 | Threonine--tRNA ligase (EC 6.1.1.3) (Threonyl-tRNA synthetase) (ThrRS) | 10 |
| 96 | *Rv1915*  *aceAa* | N([5](#_ENREF_5)) | O07718 | Probable isocitrate lyase aceAa [first part] (isocitrase) (isocitratase) (ICL) (EC 4.1.3.1) | 10 |
| 97 | *Rv0129c*  *fbpC* | N([2](#_ENREF_2)) | P0A4V4 | Antigen 85-C (Antigen 85 complex C) (85C) (Ag85C) (Fibronectin-binding protein C) (Mycolyl transferase 85C) (EC 2.3.1.-) | 9 |
| 98 | *Rv2157c*  *murF* | Y([2](#_ENREF_2)) | P0A5L4 | UDP-N-acetylmuramoyl-tripeptide--D-alanyl-D-alanine ligase (EC 6.3.2.10) (D-alanyl-D-alanine-adding enzyme) (UDP-MurNAc-pentapeptide synthetase) | 9 |
| 99 | *Rv3411c*  *guaB2* | Y([15](#_ENREF_15)) | P65167 | Inosine-5'-monophosphate dehydrogenase (IMP dehydrogenase) (IMPD) (IMPDH) (EC 1.1.1.205) | 9 |
| 100 | *Rv3313c*  *Add* | N([2](#_ENREF_2)) | P63907 | Adenosine deaminase (EC 3.5.4.4) (Adenosine aminohydrolase) | 9 |
| 101 | *Rv0467*  *icl1* | Y([2](#_ENREF_2)) | P0A5H3 | Isocitrate lyase (ICL) (Isocitrase) (Isocitratase) (EC 4.1.3.1) | 9 |
| 102 | *Rv0957*  *purH* | Y([2](#_ENREF_2)) | P67541 | Bifunctional purine biosynthesis protein PurH [Includes: Phosphoribosylaminoimidazolecarboxamide formyltransferase (EC 2.1.2.3) (AICAR transformylase); IMP cyclohydrolase (EC 3.5.4.10) (ATIC) (IMP synthase) (Inosinicase)] | 8 |
| 103 | *Rv2129c* | N([2](#_ENREF_2)) | O33263 | Probable oxidoreductase (EC 1.-.-.-) | 8 |
| 104 | *Rv3410c*  *guaB3* | Y([2](#_ENREF_2)) | P65170 | Uncharacterized oxidoreductase Rv3410c/MT3518 (EC 1.-.-.-) | 8 |
| 105 | *Rv1293*  *lysA* | Y([2](#_ENREF_2)) | P0A5M4 | Diaminopimelate decarboxylase (DAP decarboxylase) (DAPDC) (EC 4.1.1.20) | 8 |
| 106 | *Rv1703c* | N([2](#_ENREF_2)) | O33202 | Probable catechol-o-methyltransferase (EC 2.1.1.6) | 7 |
| 107 | *Rv1905c*  *aao* | N([2](#_ENREF_2)) | O07727 | Probable D-amino-acid oxidase (DAAO) (DAMOX) (DAO) (EC 1.4.3.3) | 7 |
| 108 | *Rv1547*  *dnaE1* | Y([2](#_ENREF_2)) | P63977 | DNA polymerase III subunit alpha (EC 2.7.7.7) | 6 |
| 109 | *Rv2903c*  *lepB* | Y([2](#_ENREF_2)) | Q10789 | Probable signal peptidase I (SPase I) (EC 3.4.21.89) (Leader peptidase I) | 6 |
| 110 | *Rv0050*  *ponA1* | N([2](#_ENREF_2)) | P71707 | Probable bifunctional penicillin-binding prtein 1A/1B PonA1 (Murein polymerase) (PBP1): penicillin-insensitive transglycosylase (peptidoglycan tgase + penicillin-sensitive transpeptidase (DD- transpeptidase) (EC 2.4.2.-) (EC 3.4.-.-) | 6 |
| 111 | *Rv2447c*  *folC* | Y([2](#_ENREF_2)) | O53174 | Folylpolyglutamate synthase (EC 6.3.2.17) (Probable folylpolyglutamate synthase protein FolC (folylpoly-gamma-glutamate synthetase) (FPGS)) (EC 6.3.2.17) | 6 |
| 112 | *Rv0781*  *ptrBa* | N([2](#_ENREF_2)) | P71835 | Probable protease II PtaBa [first part] (Oligopeptidase B) (EC 3.4.21.83) | 5 |
| 113 | *Rv3566c*  *nhoA* | N([2](#_ENREF_2)) | P0A5L8 | Arylamine N-acetyltransferase (EC 2.3.1.5) | 5 |
| 114 | *Rv0162c*  *adhE1* | N([2](#_ENREF_2)) | Q7DAC8 | Alcohol dehydrogenase, zinc-containing (Probable zinc-type alcohol dehydrogenase (E subunit) AdhE) (EC 1.1.1.1) | 5 |
| 115 | *Rv1449c*  *tkt* | Y([16](#_ENREF_16)) | O06811 | Transketolase (TK) (EC 2.2.1.1) | 5 |
| 116 | *Rv1206*  *fadD6* | N([2](#_ENREF_2)) | O05307 | Probable fatty-acid-CoA ligase FadD6 (fatty-acid-CoA synthetase) (fatty-acid-CoA synthase) (EC 6.2.1.-) (Very-long-chain acyl-CoA synthetase, putative) | 5 |
| 117 | *Rv3247c*  *tmk* | Y([2](#_ENREF_2)) | O05891 | Thymidylate kinase (EC 2.7.4.9) (Thymidine monophosphate kinase) (dTMP kinase) (TMPK) | 5 |
| 118 | *Rv2173*  *idsA2* | N([2](#_ENREF_2)) | O53507 | Probable geranylgeranyl pyrophosphate synthetase IdsA2 (GGPPSASE) (GGPP synthetase) (geranylgeranyl diphosphate synthase) (EC 2.5.1.-) (Polyprenyl synthetase) | 4 |
| 119 | *Rv0548c*  *menB* | Y([2](#_ENREF_2)) | O06414 | 1,4-Dihydroxy-2-naphthoyl-CoA synthase (DHNA-CoA synthase) (EC 4.1.3.36) | 3 |
| 120 | *Rv0194* | N([5](#_ENREF_5)) | O53645 | Probable drug-transport transmembrane ATP-binding protein ABC transporter | 3 |
| 121 | *Rv0761c*  *adhB* | N([2](#_ENREF_2)) | P71818 | Alcohol dehydrogenase B (EC 1.1.1.1) | 3 |
| 122 | *Rv1316c*  *adaB* | N([2](#_ENREF_2)) | P0A696 | Methylated-DNA--protein-cysteine methyltransferase (EC 2.1.1.63) (6-O-methylguanine-DNA methyltransferase) (MGMT) (O-6-methylguanine-DNA-alkyltransferase) | 3 |
| 123 | *Rv0198c*  *zmp1* | N([2](#_ENREF_2)) | O53649 | Endopeptidase, peptidase family M13 (Probable zinc metalloprotease) (EC 3.4.24.-) | 3 |
| 124 | *Rv3086*  *adhD* | N([2](#_ENREF_2)) | O53303 | Probable zinc-type alcohol dehydrogenase AdhD (aldehyde reductase) (EC 1.1.1.-) (Zinc-binding dehydrogenase) | 3 |
| 125 | *Rv1987* | N([2](#_ENREF_2)) | P64905 | Uncharacterized protein Rv1987/MT2041 | 3 |
| 126 | *Rv2540c*  *aroF* | Y([2](#_ENREF_2)) | P63611 | Chorismate synthase (EC 4.2.3.5) (5-enolpyruvylshikimate-3-phosphate phospholyase) | 3 |
| 127 | *Rv3315c*  *cdd* | N([2](#_ENREF_2)) | O53367 | Cytidine deaminase (EC 3.5.4.5) (probable cytidine deaminase CDD (cytidine aminohydrolase) (cytidine nucleoside deaminase)) (EC 3.5.4.5) | 2 |
| 128 | *Rv0809*  *purM* | Y([2](#_ENREF_2)) | O53823 | Phosphoribosylformylglycinamidine cyclo-ligase (EC 6.3.3.1) | 1 |
| 129 | *Rv0772*  *purD* | Y([2](#_ENREF_2)) | P65893 | Phosphoribosylamine--glycine ligase (EC 6.3.4.13) (GARS) (Glycinamide ribonucleotide synthetase) (Phosphoribosylglycinamide synthetase) | 1 |
| 130 | *Rv2089c*  *pepE* | N([2](#_ENREF_2)) | P65810 | Probable dipeptidase pepE (EC 3.4.13.-) | 1 |
| 131 | *Rv1844c*  *gnd1* | N([2](#_ENREF_2)) | Q79FJ2 | 6-phosphogluconate dehydrogenase, decarboxylating (EC 1.1.1.44) | 1 |
| 132 | *Rv1122*  *gnd2* | Y([2](#_ENREF_2)) | O06574 | 6-phosphogluconate dehydrogenase, decarboxylating, putative (EC 1.1.1.44)) | 1 |
| 133 | *Rv2754c*  *thyX* | Y([10](#_ENREF_10)) | P66930 | Thymidylate synthase thyX (TS) (TSase) (EC 2.1.1.148) | 1 |
| 134 | *Rv0458* | N([2](#_ENREF_2)) | P63937 | Probable aldehyde dehydrogenase (EC 1.2.1.3) | 1 |
| 135 | *Rv3856c* | N([2](#_ENREF_2)) | P96221 | Putative uncharacterized protein | 1 |
| 136 | *Rv2201*  *asnB* | Y([2](#_ENREF_2)) | P64247 | Putative asparagine synthetase [glutamine-hydrolyzing] (EC 6.3.5.4) | 1 |
| 137 | *Rv3307*  *deoD* | N([2](#_ENREF_2)) | P0A538 | Purine nucleoside phosphorylase (PNP) (EC 2.4.2.1) (Inosine phosphorylase) | 1 |

* - Essentiality was attributed by first consulting any *in vivo* data when available, then by looking at individual knock outs *in vitro* and finally using information from *in vitro* transposon mutagenesis studies.

**Table S2b:** Number of compounds for each predicted *M. tuberculosis* target using SEA. Targets are ranked in descending order of number of assigned ligands

|  | *M. tuberculosis*  *Gene id* | Essentiality* | *M. tuberculosis*  UniProt Accession | *M. tuberculosis* Protein Name | Number of compounds |
| --- | --- | --- | --- | --- | --- |
| 1 | *Rv0404*  *fadD30* | N([2](#_ENREF_2)) | P95213 | Long-chain-fatty-acid-AMP ligase FadD30 (EC 6.2.1.-) (Acyl-AMP synthetase) | 77 |
| 2 | *Rv3170*  *aofH* | N([2](#_ENREF_2)) | P63533 | Putative flavin-containing monoamine oxidase AofH (EC 1.4.3.-) | 69 |
| 3 | *Rv2505c*  *fadD35* | N([2](#_ENREF_2)) | O06168 | Probable Fatty-Acid-CoA Ligase FADD35 (Fatty-Acid-CoA synthase) (EC 6.2.1.-) (Substrate-CoA ligase) | 68 |
| 4 | *Rv0435c* | N(2) | P96281 | Cell division control protein, putative (putative conserved ATPase) (EC 3.6.1.-) | 55 |
| 5 | *Rv3617*  *ephA* | N(6) | O06266 | Epoxide hydrolase (EC 3.3.2.9) (probable epoxide hydrolase EphA (epoxide hydratase) (arene-oxide hydratase)) (EC 3.3.2.3) | 54 |
| 6 | *Rv3089*  *fadD13* | N(2) | O53306 | Probable Fatty-Acid-CoA Ligase FADD13 (Fatty-Acid-CoA synthase) (EC 6.2.1.-) (Substrate-CoA ligase) | 48 |
| 7 | *Rv0533c*  *fabH* | N(2) | P0A574 | 3-oxoacyl-[acyl-carrier-protein] synthase 3 (EC 2.3.1.180) (3-oxoacyl-[acyl-carrier-protein] synthase III) (Beta-ketoacyl-ACP synthase III) (KAS III) (MtFabH) | 45 |
| 8 | *Rv3411c*  *guaB2* | Y([15](#_ENREF_15)) | P65167 | Inosine-5'-monophosphate dehydrogenase (IMP dehydrogenase) (IMPD) (IMPDH) (EC 1.1.1.205) | 38 |
| 9 | *Rv0270*  *fadD2* | N(2) | P95227 | Probable Fatty-Acid-CoA Ligase FADD2 (Fatty-Acid-CoA synthase) (EC 6.2.1.-) (Substrate-CoA ligase) | 37 |
| 10 | *Rv2045c*  *lipT* | N(2) | O53488 | Probable carboxylesterase LipT (EC 3.1.1.-) | 35 |
| 11 | *Rv0183* | N(2) | O07427 | POSSIBLE LYSOPHOSPHOLIPASE (EC 3.1.-.-) | 29 |
| 12 | *Rv0119*  *fadD7* | N(2) | O07169 | Probable Fatty-Acid-CoA Ligase FADD7 (Fatty-Acid-CoA synthase) (EC 6.2.1.-) (Substrate-CoA ligase) | 28 |
| 13 | *Rv3588c*  *canB* | Y([4](#_ENREF_4)) | O53573 | Carbonic anhydrase (EC 4.2.1.1) (Carbonate dehydratase) | 27 |
| 14 | *Rv2139*  *pyrD* | Y(2) | P65908 | Dihydroorotate dehydrogenase (quinone) (EC 1.3.5.2) (DHOdehase) (DHOD) (DHODase) (Dihydroorotate oxidase) | 27 |
| 15 | *Rv3410c*  *guaB3* | Y(2) | P65170 | Uncharacterized oxidoreductase Rv3410c/MT3518 (EC 1.-.-.-) | 26 |
| 16 | *Rv1747* | Y([12](#_ENREF_12)) | O65934 | Probable conserved transmembrane ATP-binding protein ABC transporter | 25 |
| 17 | *Rv0194* | N([5](#_ENREF_5)) | O53645 | Probable drug-transport transmembrane ATP-binding protein ABC transporter | 23 |
| 18 | *Rv2763c*  *dfrA* | Y(2) | P0A546 | Dihydrofolate reductase (EC 1.5.1.3) | 21 |
| 19 | *Rv3512* | N(2) | Q6MWW7 | PE-PGRS Family protein | 21 |
| 20 | *Rv0781*  *ptrBa* | N(2) | P71835 | Probable protease II PtaBa [first part] (Oligopeptidase B) (EC 3.4.21.83) | 18 |
| 21 | *Rv1484*  *inhA* | Y(2) | P0A5Y6 | Enoyl-[acyl-carrier-protein] reductase [NADH] (EC 1.3.1.9) (NADH-dependent enoyl-ACP reductase) | 17 |
| 22 | *Rv0166*  *fadD5* | N(2) | O07411 | Probable Fatty-Acid-CoA Ligase FADD5 (Fatty-Acid-CoA synthase) (EC 6.2.1.-) (Substrate-CoA ligase) | 17 |
| 23 | *Rv0429c*  *Def* | Y(2) | P96275 | Peptide deformylase (PDF) (EC 3.5.1.88) (Polypeptide deformylase) | 15 |
| 24 | *Rv1640c*  *lysX* | Y([6](#_ENREF_6)) | P94974 | Lysylphosphatidylglycerol biosynthesis bifunctional protein LysX [Includes: Lysine--tRNA ligase (EC 6.1.1.6) (Lysyl-tRNA synthetase) (LysRS); Phosphatidylglycerol lysyltransferase (EC 2.3.2.3) (Lysylphosphatidylglycerol synthetase) (LPG synthetase)] | 15 |
| 25 | *Rv2443*  *dctA* | N(2) | P71906 | C4-dicarboxylate transport protein (probable C4-dicarboxylate-transport transmembrane protein Dcta) | 15 |
| 26 | *Rv2764c*  *thyA* | N([10](#_ENREF_10)) | P67044 | Thymidylate synthase (TS) (TSase) (EC 2.1.1.45) | 15 |
| 27 | *Rv2971* | Y(2) | P95124 | Uncharacterized oxidoreductase Rv2971/MT3049 (EC 1.-.-.-) | 15 |
| 28 | *Rv2483c*  *plsC* | Y([4](#_ENREF_4)) | O53208 | Acyltransferase family protein | 13 |
| 29 | *Rv3273* | N(2) | P96878 | Probable transmembrane carbonic anhydrase (carbonate dehydratase) (carbonic dehydratase) (EC 4.2.1.1) (Sulfate transporter/carbonic anhydrase, putative) | 13 |
| 30 | *Rv3569*  *hsaD* | Y(6) | P96851 | 4,5-9,10-diseco-3-hydroxy-5,9,17-trioxoandrosta-1(10),2-diene-4-oate hydrolase (2-hydroxy-6-oxo-6-phenylhexa-2,4-dienoate hydrolase) (HOPDA hydrolase) (EC 3.7.1.8) (Meta-cleavage product hydrolase) (MCP hydrolase) | 13 |
| 31 | *Rv3561*  *fadD3* | N(2) | P96843 | Probable Fatty-Acid-CoA Ligase FADD3 (Fatty-Acid-CoA synthase) (EC 6.2.1.-) (Substrate-CoA ligase) | 12 |
| 32 | *Rv2129c* | N(2) | O33263 | Probable oxidoreductase (EC 1.-.-.-) | 12 |
| 33 | *Rv2855*  *Mtr* | N(2) | O07927 | Mycothione reductase (EC 1.8.1.15) (Mycothiol-disulfide reductase) (NADPH-dependent mycothione reductase) | 12 |
| 34 | *Rv1599*  *hisD* | Y(2) | P63950 | Histidinol dehydrogenase (HDH) (EC 1.1.1.23) | 11 |
| 35 | *Rv2182c* | Y(2) | O53516 | 1-acylglycerol-3-phosphate O-acyltransferase (EC 2.3.1.51) (Acyltransferase family protein) | 11 |
| 36 | *Rv3598c*  *lysS* | Y(2) | P67607 | Lysine--tRNA ligase 1 (EC 6.1.1.6) (Lysyl-tRNA synthetase 1) (LysRS 1) | 11 |
| 37 | *Rv2361c*  *uppS* | Y(2) | P60479 | Undecaprenyl pyrophosphate synthase (UPP synthase) (EC 2.5.1.31) (Di-trans,poly-cis-decaprenylcistransferase) (Ditrans,polycis-undecaprenyl-diphosphate synthase) (Undecaprenyl diphosphate synthase) (UDS) | 10 |
| 38 | *Rv2234*  *ptpA* | Y([1](#_ENREF_1)) | P65716 | Probable low molecular weight protein-tyrosine-phosphatase (PTPase) (EC 3.1.3.48) | 10 |
| 39 | *Rv0764c*  *cyp51* | N(2) | P0A512 | Lanosterol 14-alpha demethylase (EC 1.14.13.70) (CYPLI) (Cytochrome P450 51) (Cytochrome P450-14DM) (Cytochrome P450-LIA1) (Sterol 14-alpha demethylase) | 10 |
| 40 | *Rv1086* | N(2) | O53434 | Short-chain Z-isoprenyl diphosphate synthase (EC 2.5.1.68) ((2Z,6E)-farnesyl diphosphate synthase) (Z-FPP synthase) (Z-FPPS) (Z-isoprenyl diphosphate synthase) | 9 |
| 41 | *Rv1284*  *canA* | Y([4](#_ENREF_4)) | P64797 | Putative carbonate dehydratase-like protein Rv1284 (EC 4.2.1.-) | 9 |
| 42 | *Rv1730c* | N(2) | P71988 | Possible penicillin-binding protein | 9 |
| 43 | *Rv1707* | N(2) | O33206 | Probable conserved transmembrane protein (Sulfate transporter) | 9 |
| 44 | *Rv0373c* | N(2) | O53708 | Carbon monoxide dehydrogenase, large subunit, putative (probable carbon monoxide dehydrogenase (large chain)) (EC 1.2.99.2) | 9 |
| 45 | *Rv2781c* | N([4](#_ENREF_4)) | O33323 | 2-nitropropane dioxygenase, putative (possible alanine rich oxireductase) (EC 1.-.-.-) | 8 |
| 46 | *Rv3218* | N(2) | O05848 | Diacylglycerol kinase catalytic domain-containing protein | 8 |
| 47 | *Rv1899c*  *lppD* | N(2) | O07733 | Possible lipoprotein LppD | 8 |
| 48 | *Rv3553* | N([4](#_ENREF_4)) | P71847 | Possible oxireductase (EC 1.-.-.-) | 7 |
| 49 | *Rv3397c*  *phyA* | N([4](#_ENREF_4)) | P65860 | Probable phytoene synthase (EC 2.5.1.32) | 7 |
| 50 | *Rv0058*  *dnaB* | Y(2) | P71715 | Replicative DNA helicase DnaB (EC 3.6.4.12) | 7 |
| 51 | *Rv3825c*  *pks2* | N(2) | O07798 | Phthioceranic/hydroxyphthioceranic acid synthase (EC 2.3.1.-) (Polyketide synthase pks2) | 6 |
| 52 | *Rv0482*  *murB* | Y(2) | P65460 | UDP-N-acetylenolpyruvoylglucosamine reductase (EC 1.1.1.158) (UDP-N-acetylmuramate dehydrogenase) | 6 |
| 53 | *Rv1987* | N(2) | P64905 | Uncharacterized protein Rv1987/MT2041 | 6 |
| 54 | *Rv0198c*  *zmp1* | N(2) | O53649 | Endopeptidase, peptidase family M13 (Probable zinc metalloprotease) (EC 3.4.24.-) | 6 |
| 55 | *Rv2766c*  *fabG5* | N(2) | Q7D6M3 | Oxidoreductase, short-chain dehydrogenase/reductase family (probable short-chain type dehydrogenase/reductase) (EC 1.-.-.-) | 5 |
| 56 | *Rv1601*  *hisB* | Y(2) | P64368 | Imidazoleglycerol-phosphate dehydratase (IGPD) (EC 4.2.1.19) | 5 |
| 57 | *Rv3627c* | Y(2) | O06380 | Putative uncharacterized protein | 5 |
| 58 | *Rv3855*  *ethR* | N(2) | P96222 | HTH-type transcriptional regulator EthR | 5 |
| 59 | *Rv2947c*  *pks15* | N(2) | P96284 | Putative inactive phenolphthiocerol synthesis polyketide synthase type I Pks15 | 5 |
| 60 | *Rv2299c*  *htpG* | N(2) | P64411 | Chaperone protein htpG (Heat shock protein htpG) (High temperature protein G) | 5 |
| 61 | *Rv1703c* | N(2) | O33202 | Probable catechol-o-methyltransferase (EC 2.1.1.6) | 4 |
| 62 | *Rv2157c*  *murF* | Y(2) | P0A5L4 | UDP-N-acetylmuramoyl-tripeptide--D-alanyl-D-alanine ligase (EC 6.3.2.10) (D-alanyl-D-alanine-adding enzyme) (UDP-MurNAc-pentapeptide synthetase) | 4 |
| 63 | *Rv2110c*  *prcB* | Y([13](#_ENREF_13)) | O33245 | Proteasome subunit beta (EC 3.4.25.1) (20S proteasome beta subunit) (Proteasome core protein PrcB) | 4 |
| 64 | *Rv0957*  *purH* | Y(2) | P67541 | Bifunctional purine biosynthesis protein PurH [Includes: Phosphoribosylaminoimidazolecarboxamide formyltransferase (EC 2.1.2.3) (AICAR transformylase); IMP cyclohydrolase (EC 3.5.4.10) (ATIC) (IMP synthase) (Inosinicase)] | 4 |
| 65 | *Rv3602c*  *panC* | N([14](#_ENREF_14)) | P0A5R0 | Pantothenate synthetase (PS) (EC 6.3.2.1) (Pantoate--beta-alanine ligase) (Pantoate-activating enzyme) | 4 |
| 66 | *Rv1315*  *murA* | Y(2) | P0A5L2 | UDP-N-acetylglucosamine 1-carboxyvinyltransferase (EC 2.5.1.7) (Enoylpyruvate transferase) (UDP-N-acetylglucosamine enolpyruvyl transferase) (EPT) | 3 |
| 67 | *Rv3525c* | N(2) | P71876 | Possible siderophore-binding protein | 3 |
| 68 | *Rv2068c*  *blaC* | N(2) | P0C5C1 | Beta-lactamase (EC 3.5.2.6) (Penicillinase) | 3 |
| 69 | *Rv2523c*  *acpS* | N(2) | P0A4W8 | Holo-[acyl-carrier-protein] synthase (Holo-ACP synthase) (EC 2.7.8.7) (4'-phosphopantetheinyl transferase AcpS) | 3 |
| 70 | *Rv0091*  *Mtn* | N(2) | P67656 | MTA/SAH nucleosidase (EC 3.2.2.9) (5'-methylthioadenosine nucleosidase) (S-adenosylhomocysteine nucleosidase) | 3 |
| 71 | *Rv2200c*  *ctaC* | Y(2) | P63854 | Cytochrome c oxidase subunit 2 (EC 1.9.3.1) | 3 |
| 72 | *Rv2210c*  *ilvE* | Y(2) | Q10399 | Branched-chain-amino-acid aminotransferase (BCAT) (EC 2.6.1.42) | 3 |
| 73 | *Rv0458* | N(2) | P63937 | Probable aldehyde dehydrogenase (EC 1.2.1.3) | 2 |
| 74 | *Rv2121c*  *hisG* | Y(2) | P60759 | ATP phosphoribosyltransferase (ATP-PRT) (ATP-PRTase) (EC 2.4.2.17) | 2 |
| 75 | *Rv1323*  *fadA4* | Y([4](#_ENREF_4)) | P66926 | Probable acetyl-CoA acetyltransferase (EC 2.3.1.9) (Acetoacetyl-CoA thiolase) | 2 |
| 76 | *Rv0427c*  *xthA* | Y([4](#_ENREF_4)) | P96273 | Exodeoxyribonuclease III (EC 3.1.11.2) | 2 |
| 77 | *Rv3608c*  *folP1* | Y(2) | P0A578 | Dihydropteroate synthase 1 (DHPS 1) (EC 2.5.1.15) (Dihydropteroate pyrophosphorylase 1) | 2 |
| 78 | *Rv1629*  *polA* | Y(2) | P0A550 | DNA polymerase I (POL I) (EC 2.7.7.7) | 2 |
| 79 | *Rv2946c*  *pks1* | N(2) | P96285 | synthase type I Pks1 | 2 |
| 80 | *Rv3197* | N(2) | O53343 | Probable conserved ATP-Binding Protein ABC transporter | 2 |
| 81 | *Rv3255c*  *manA* | Y(2) | O05898 | Mannose-6-phosphate isomerase (EC 5.3.1.8) | 2 |
| 82 | *Rv1338*  *murI* | Y([11](#_ENREF_11)) | P63635 | Glutamate racemase (EC 5.1.1.3) | 2 |
| 83 | *Rv1436*  *gap* | Y(2) | P64178 | Glyceraldehyde-3-phosphate dehydrogenase (GAPDH) (EC 1.2.1.12) | 2 |
| 84 | *Rv2845c*  *proS* | Y(2) | O05814 | Proline-tRNA ligase (EC 6.1.1.15) Prolyl-tRNA synthetase | 2 |
| 85 | *Rv1649*  *pheS* | Y(2) | P94984 | Phenylalanine--tRNA ligase alpha subunit (EC 6.1.1.20) (Phenylalanyl-tRNA synthetase alpha subunit) (PheRS) | 2 |
| 85 | *Rv0005*  *gyrB* | Y(2) | P0C5C5 | DNA gyrase subunit B (EC 5.99.1.3) | 2 |
| 86 | *Rv3566c*  *nhoA* | N(2) | P0A5L8 | Arylamine N-acetyltransferase (EC 2.3.1.5) | 1 |
| 88 | *Rv3313c*  *Add* | N(2) | P63907 | Adenosine deaminase (EC 3.5.4.4) (Adenosine aminohydrolase) | 1 |
| 89 | *Rv0800*  *pepC* | N(2) | O06634 | Probable M18 family aminopeptidase 2 (EC 3.4.11.-) | 1 |
| 90 | *Rv2701c*  *suhB* | N(2) | P65165 | Inositol-1-monophosphatase SuhB (I-1-Pase) (IMPase) (Inositol-1-phosphatase) (EC 3.1.3.25) | 1 |
| 91 | *Rv1547*  *dnaE1* | Y(2) | P63977 | DNA polymerase III subunit alpha (EC 2.7.7.7) | 1 |
| 92 | *Rv1316c*  *adaB* | N(2) | P0A696 | Methylated-DNA--protein-cysteine methyltransferase (EC 2.1.1.63) (6-O-methylguanine-DNA methyltransferase) (MGMT) (O-6-methylguanine-DNA-alkyltransferase) | 1 |
| 93 | *Rv0809*  *purM* | Y(2) | O53823 | Phosphoribosylformylglycinamidine cyclo-ligase (EC 6.3.3.1) | 1 |
| 94 | *Rv3307*  *deoD* | N(2) | P0A538 | Purine nucleoside phosphorylase (PNP) (EC 2.4.2.1) (Inosine phosphorylase) | 1 |
| 95 | *Rv2540c*  *aroF* | Y(2) | P63611 | Chorismate synthase (EC 4.2.3.5) (5-enolpyruvylshikimate-3-phosphate phospholyase) | 1 |
| 96 | *Rv0772*  *purD* | Y(2) | P65893 | Phosphoribosylamine--glycine ligase (EC 6.3.4.13) (GARS) (Glycinamide ribonucleotide synthetase) (Phosphoribosylglycinamide synthetase) | 1 |
| 97 | *Rv2384*  *mbtA* | N(2) | P71716 | 2,3-dihydroxybenzoate-AMP ligase (bifunctional enzyme Mbta: salicyl-AMP ligase (SAL-AMP ligase) + salicyl-S-ArCP synthetase) (EC 6.-.-.-) | 1 |
| 98 | *Rv1850*  *ureC* | N(2) | P0A660 | Urease subunit alpha (EC 3.5.1.5) (Urea amidohydrolase subunit alpha) | 1 |
| 99 | *Rv1872c*  *lldD2* | N(2) | P95143 | Putative L-lactate dehydrogenase [cytochrome] (EC 1.1.2.3) | 1 |
| 100 | *Rv3086*  *adhD* | N(2) | O53303 | Probable zinc-type alcohol dehydrogenase AdhD (aldehyde reductase) (EC 1.1.1.-) (Zinc-binding dehydrogenase) | 1 |
| 101 | *Rv0162c*  *adhE1* | N(2) | Q7DAC8 | Alcohol dehydrogenase, zinc-containing (Probable zinc-type alcohol dehydrogenase (E subunit) AdhE) (EC 1.1.1.1) | 1 |
| 102 | *Rv0761c*  *adhB* | N(2) | P71818 | Alcohol dehydrogenase B (EC 1.1.1.1) | 1 |
| 103 | *Rv1007c*  *metS* | Y(2) | O05593 | Methionine--tRNA ligase (EC 6.1.1.10) (Methionyl-tRNA synthetase) (MetRS) | 1 |
| 104 | *Rv2447c*  *folC* | Y(2) | O53174 | Folylpolyglutamate synthase (EC 6.3.2.17) (Probable folylpolyglutamate synthase protein FolC (folylpoly-gamma-glutamate synthetase) (FPGS)) (EC 6.3.2.17) | 1 |
| 105 | *Rv1905c*  *aao* | N(2) | O07727 | Probable D-amino-acid oxidase (DAAO) (DAMOX) (DAO) (EC 1.4.3.3) | 1 |
| 106 | *Rv2467*  *pepD* | N(2) | Q7D736 | Probable serine protease PepD (serine proteinase) (EC 3.4.21.-) | 1 |
| 107 | *Rv2332*  *mez* | N(2) | P71880 | Putative malate oxidoreductase [NAD] (EC 1.1.1.38) (Malic enzyme) | 1 |
| 108 | *Rv0211*  *pckA* | N(2) | P65686 | Phosphoenolpyruvate carboxykinase [GTP] (PEP carboxykinase) (PEPCK) (EC 4.1.1.32) (Phosphoenolpyruvate carboxylase) | 1 |
| 109 | *Rv2173*  *idsA2* | N(2) | O53507 | Probable geranylgeranyl pyrophosphate synthetase IdsA2 (GGPPSASE) (GGPP synthetase) (geranylgeranyl diphosphate synthase) (EC 2.5.1.-) (Polyprenyl synthetase) | 1 |
| 110 | *Rv2870c*  *Dxr* | Y([9](#_ENREF_9)) | P64012 | 1-deoxy-D-xylulose 5-phosphate reductoisomerase (DXP reductoisomerase) (EC 1.1.1.267) (1-deoxyxylulose-5-phosphate reductoisomerase) (2-C-methyl-D-erythritol 4-phosphate synthase) | 1 |

* - Essentiality was attributed by first consulting any *in vivo* data when available, then by looking at individual knock outs *in vitro* and finally using information from *in vitro* transposon mutagenesis studies.

**Table S3**: Target prediction scores, EthR inhibition values and structural information for the *in vitro* assayed 35 compounds

| Compound | SPR  Inhibition (%) | SPR  IC_50_ (µM) | EthR  Zscore E-value LEI | | | InhA  Zscore E-value LEI | | |
| --- | --- | --- | --- | --- | --- | --- | --- | --- |
|   GSK1107112a  (1) | 100 | 12 | nd | nd | 1.58 | nd | nd | 1.11 |
|   GSK1570606a  (2) | 100 | 9.9 | 1.45 | nd | 1.17 | nd | nd | 1.26 |
|   GSK2032710a  (3) | 100 | 3.9 | 1.4 |  | 0.78 | nd | nd | 0.72 |
|   GSK735826a  (4) | 100 | 13 | 1.57 | nd | 1.24 |  |  | 1.13 |
|   GSK445886a  (5) | 99 | 30 | nd | nd | 1.55 | nd | nd | 1.24 |
|   GSK735816a  (6) | 97 | 22 | 1.95 | nd | 1.35 | 4.6 | 1.98E-03 | 1.28 |
|   GSK920684a  (7) | 93 | 50 | 2.71 | nd | 1.17 | nd | nd | 1.15 |
| 3  GSK1742694a  (8) | 93 | ND | nd | nd | 1.29 | nd | nd | 1.15 |
|   GSK1372568a  (9) | 80 | ND | 1.54 | nd | 1.42 | nd | nd | 1.19 |
|   GSK921295a  (10) | 74 | >100 | nd | nd | 1.31 | nd | nd | 1.09 |
|   GSK690382a  (11) | 72 | ND | 1.72 | nd | 1.39 | 3.71 | 128E-05 | 1.17 |
|   SB-435634  (12) | 67 | >100 | nd | nd | 1.18 | nd | nd | 1.41 |
|   GSK1365028a  (13) | 62 | ND | nd | nd | 1.35 | 1.67 | nd | 0.98 |
|   GSK1733953a  (14) | 62 | ND | nd | nd | 0.76 | nd | nd | 0.84 |
|   GR135486x  (15) | 61 | ND | nd | nd | 1.52 | nd | nd | 1.15 |
|   GSK888636a  (16) | 60 | ND | nd | nd | 1.05 | nd | nd | 0.69 |
|   GSK381407a  (17) | 59 | ND | nd | nd | 0.83 | nd | nd | Nd |
|   SB-204804-a  (18) | 59 | ND | nd | nd | 0.6 | nd | nd | nd |
|   GSK2157753a  (19) | 58 | 45 | 2.25 | nd | 1.65 | nd | nd | 1.5 |
|   GSK1731114a  (20) | 56 | ND | nd | nd | 0.73 | nd | nd | Nd |
|   GSK270670a  (21) | 54 | ND | nd | nd | 0.66 | nd | nd | 0.5 |
|   GW356807a  (22) | 54 | ND | nd | nd | 0.94 | nd | nd | Nd |
|   GSK463114a  (23) | 54 | ND | nd | nd | 0.88 | nd | nd | 0.94 |
|   GSK1402290a  (24) | 51 | ND | nd | nd | 1.15 | nd | nd | Nd |
|   GSK1302651a  (25) | 50 | ND | nd | nd | 1.19 | nd | nd | 1.12 |
|   GR135487x  (26) | 47 | ND | nd | nd | 1.36 | nd | nd | 1.47 |
|   GSK1812410a  (27) | 45 | ND | nd | nd | 0.58 | nd | nd | Nd |
|   GSK358607a  (28) | 42 | ND | 1.53 | nd | 0.78 | nd | nd | 0.75 |
|   GV187303x  (29) | 42 | ND | nd | nd | 1.26 | 1.77 | 4.19E-07 | 1.46 |
|   BRL-51091am  (30) | 41 | ND | nd | nd | 0.72 | 2.50 | 5.67E-21 | 0.56 |
|   GSK353069a  (31) | 38 | ND | nd | nd | 1.08 | nd | nd | 1.13 |
|   GSK353496a  (32) | 33 | ND | 1.42 | nd | 1.30 | nd | nd | 1.72 |
|   GSK3011724a  (33) | 28 | ND | nd | nd | 1.35 | nd | nd | 1.16 |
|   GSK957094a  (34) | 27 | ND | nd | nd | 1.07 | nd | nd | 1.23 |
|   brl-10988sa  (35) | 24 | ND | nd | nd | 0.44 | nd | nd | 2.74 |

- ND – IC_50_ not determined, nd – not detected

**Table S4**: List of other predicted targets for the 8 EthR hits

| GSKnumber | Z_score | *M. tuberculosis* Protein | Essentiality* |
| --- | --- | --- | --- |
| SB-435634  (7 targets)  Compound 12 | 2.18 | Rv2443  DctA | N([2](#_ENREF_2)) |
|  | 1.93 | Rv3588c  CanB | Y([4](#_ENREF_4)) |
|  | 1.76 | Rv1599  HisD | Y([2](#_ENREF_2)) |
|  | 1.60 | Rv0363c  Fba | Y([2](#_ENREF_2)) |
|  | 1.55 | Rv1981c  NrdF1 | N([2](#_ENREF_2)) |
|  | 1.55 | Rv2737c  RecA | N([8](#_ENREF_8)) |
|  | 1.53 | Rv2384  MbtA | N([2](#_ENREF_2)) |
| GSK2157753A  (24)  compound 19 | 3.06 | Rv2933  PpsC | N([2](#_ENREF_2)) |
|  | 3.06 | Rv2121c  HisG | Y([2](#_ENREF_2)) |
|  | 2.25 | Rv2940c  Mas | N([2](#_ENREF_2)) |
|  | 2.25 | Rv3855  EthR | N([2](#_ENREF_2)) |
|  | 2.18 | Rv3800  Pks13 | Y([2](#_ENREF_2)) |
|  | 2.18 | Rv0429c  Def | Y([2](#_ENREF_2)) |
|  | 2.05 | Rv2934  PpsD | N([2](#_ENREF_2)) |
|  | 2.05 | Rv0734  MapA | Y([3](#_ENREF_3)) |
|  | 1.96 | Rv2045c  LipT | N([2](#_ENREF_2)) |
|  | 1.96 | Rv1661  Pks7 | N([2](#_ENREF_2)) |
|  | 1.86 | Rv2234  PtpA | Y([1](#_ENREF_1)) |
|  | 1.81 | *Rv1180*  Pks3 | N([2](#_ENREF_2)) |
|  | 1.81 | Rv0482  MurB | Y([2](#_ENREF_2)) |
|  | 1.76 | Rv1527c  Pks5 | N([2](#_ENREF_2)) |
|  | 1.76 | Rv2210c  IlvE | Y([2](#_ENREF_2)) |
|  | 1.73 | Rv1181  Pks4 | N([2](#_ENREF_2)) |
|  | 1.73 | Rv1436  Gap | Y([2](#_ENREF_2)) |
|  | 1.69 | Rv0427c  xthA | Y([4](#_ENREF_4)) |
|  | 1.69 | Rv2048c  Pks12 | Y([4](#_ENREF_4)) |
|  | 1.66 | Rv2737c  RecA | N([8](#_ENREF_8)) |
|  | 1.62 | Rv2068c  BlaC | N([2](#_ENREF_2)) |
|  | 1.62 | Rv1662  Pks8 | N([2](#_ENREF_2)) |
|  | 1.59 | Rv3062  LigB | N([2](#_ENREF_2)) |
|  | 1.59 | Rv0405  Pks6 | N([5](#_ENREF_5)) |
| GSK1570606A  (8 targets)  Compound 2 | 2.86 | Rv2781c | N([4](#_ENREF_4)) |
|  | 2.48 | Rv3553 | N([4](#_ENREF_4)) |
|  | 2.25 | Rv1599  HisD | Y([2](#_ENREF_2)) |
|  | 1.79 | Rv1323  FadA4 | Y([4](#_ENREF_4)) |
|  | 1.74 | Rv2045c  LipT | N([2](#_ENREF_2)) |
|  | 1.63 | Rv0734  MapA | Y([3](#_ENREF_3)) |
|  | 1.63 | Rv3855  EthR | N([2](#_ENREF_2)) |
|  | 1.54 | Rv2121c  HisG | Y([2](#_ENREF_2)) |
| GSK921295A  (15 targets)  Compound 10 | 3.35 | Rv2781c | N([4](#_ENREF_4)) |
|  | 3.35 | Rv2931  PpsA | N([2](#_ENREF_2)) |
|  | 3.35 | Rv1662  Pks8 | N([2](#_ENREF_2)) |
|  | 2.93 | Rv3553 | N([4](#_ENREF_4)) |
|  | 2.93 | Rv0405  Pks6 | N([5](#_ENREF_5)) |
|  | 2.93 | Rv2932  PpsB | N([2](#_ENREF_2)) |
|  | 2.55 | Rv0435c | N([2](#_ENREF_2)) |
|  | 2.55 | Rv2933  PpsC | N([2](#_ENREF_2)) |
|  | 2.10 | Rv2934  PpsD | N([2](#_ENREF_2)) |
|  | 2.10 | Rv2121c  HisG | Y([2](#_ENREF_2)) |
|  | 1.92 | Rv1181  Pks4 | N([2](#_ENREF_2)) |
|  | 1.92 | Rv2947c  Pks15 | N([2](#_ENREF_2)) |
|  | 1.92 | Rv0734  MapA | Y([3](#_ENREF_3)) |
|  | 1.72 | Rv2935  PpsE | N([2](#_ENREF_2)) |
|  | 1.72 | Rv2045c  LipT | N([2](#_ENREF_2)) |
| GSK920684A  (12)  Compound 7 | 2.79 | Rv1181  Pks4 | N([2](#_ENREF_2)) |
|  | 2.79 | Rv0734  MapA | Y([3](#_ENREF_3)) |
|  | 2.79 | Rv2931  PpsA | N([2](#_ENREF_2)) |
|  | 2.71 | Rv0435c | N([2](#_ENREF_2)) |
|  | 2.71 | Rv1180  Pks3 | N([2](#_ENREF_2)) |
|  | 2.71 | Rv3855  EthR | N([2](#_ENREF_2)) |
|  | 2.71 | Rv2947c  Pks15 | N([2](#_ENREF_2)) |
|  | 2.68 | Rv2048c  Pks12 | Y([4](#_ENREF_4)) |
|  | 2.68 | Rv2861c  MapB | N([3](#_ENREF_3)) |
|  | 2.68 | Rv2932  PpsB | N([2](#_ENREF_2)) |
|  | 2.54 | Rv2781c | N([4](#_ENREF_4)) |
|  | 2.54 | Rv1662  Pks8 | Y([2](#_ENREF_2)) |
|  | 2.54 | Rv2933  PpsC | N([2](#_ENREF_2)) |
| GSK735826A  (6 targets)  Compound 4 | 3.26 | Rv0734  MapA | Y([3](#_ENREF_3)) |
|  | 2.70 | Rv2861c  MapB | N([3](#_ENREF_3)) |
|  | 1.98 | Rv2121c  HisG | Y([2](#_ENREF_2)) |
|  | 1.57 | Rv3855  EthR | N([2](#_ENREF_2)) |
|  | 1.95 | Rv2781c | N([4](#_ENREF_4)) |
|  | 1.57 | Rv0435c | N([2](#_ENREF_2)) |
| GSK735816A  (7 targets)  Compound 6 | 4.60 | Rv2121c  HisG | Y([2](#_ENREF_2)) |
|  | 2.71 | Rv0734  MapA | Y([3](#_ENREF_3)) |
|  | 2.45 | Rv2781c | N([4](#_ENREF_4)) |
|  | 2.44 | Rv2861c  MapB | N([3](#_ENREF_3)) |
|  | 2.21 | Rv2971 | Y([2](#_ENREF_2)) |
|  | 1.95 | Rv3855  EthR | N([2](#_ENREF_2)) |
|  | 1.87 | Rv3553 | N([4](#_ENREF_4)) |
|  | 1.68 | Rv0435c | N([2](#_ENREF_2)) |
| GSK445886A  (6 targets)  Compound 5 | 3.29 | Rv2045c  LipT | N([2](#_ENREF_2)) |
|  | 3.29 | Rv0435c | N([2](#_ENREF_2)) |
|  | 2.49 | Rv3170  AofH | N([2](#_ENREF_2)) |
|  | 2.49 | Rv2781c | N([4](#_ENREF_4)) |
|  | 2.45 | Rv3553 | N([4](#_ENREF_4)) |
|  | 1.89 | Rv0734  MapA | Y([3](#_ENREF_3)) |

* - Essentiality was attributed by first consulting any *in vivo* data when available, then by looking at individual knock outs *in vitro* and finally using information from *in vitro* transposon mutagenesis studies.

**Figure S1:** Omit maps” for each of ligands contoured at 1.5σ. These maps were generated using the phases from the final model.

**Figure S2:** Superposition of X-ray crystal structures of compound X in green and two published EthR inhibitors (PDB codes: 4M3B in yellow and 5EYR in orange). The distance of the ketone groups of each compound to N179 in shown in (Å)

**References**

1. H. Bach, K. G. Papavinasasundaram, D. Wong, Z. Hmama and Y. Av-Gay: Mycobacterium tuberculosis virulence is mediated by PtpA dephosphorylation of human vacuolar protein sorting 33B. *Cell Host Microbe*, 3(5), 316-22 (2008) doi:10.1016/j.chom.2008.03.008

2. M. A. DeJesus, E. R. Gerrick, W. Xu, S. W. Park, J. E. Long, C. C. Boutte, E. J. Rubin, D. Schnappinger, S. Ehrt, S. M. Fortune, C. M. Sassetti and T. R. Ioerger: Comprehensive Essentiality Analysis of the Mycobacterium tuberculosis Genome via Saturating Transposon Mutagenesis. *MBio*, 8(1) (2017) doi:10.1128/mBio.02133-16

3. O. Olaleye, T. R. Raghunand, S. Bhat, J. He, S. Tyagi, G. Lamichhane, P. Gu, J. Zhou, Y. Zhang, J. Grosset, W. R. Bishai and J. O. Liu: Methionine aminopeptidases from Mycobacterium tuberculosis as novel antimycobacterial targets. *Chem Biol*, 17(1), 86-97 (2010) doi:10.1016/j.chembiol.2009.12.014

4. C. M. Sassetti and E. J. Rubin: Genetic requirements for mycobacterial survival during infection. *Proc Natl Acad Sci U S A*, 100(22), 12989-94 (2003) doi:10.1073/pnas.2134250100

2134250100 [pii]

5. A. G. Tsolaki, A. E. Hirsh, K. DeRiemer, J. A. Enciso, M. Z. Wong, M. Hannan, Y. O. Goguet de la Salmoniere, K. Aman, M. Kato-Maeda and P. M. Small: Functional and evolutionary genomics of Mycobacterium tuberculosis: insights from genomic deletions in 100 strains. *Proc Natl Acad Sci U S A*, 101(14), 4865-70 (2004) doi:10.1073/pnas.0305634101

6. J. Rengarajan, B. R. Bloom and E. J. Rubin: Genome-wide requirements for Mycobacterium tuberculosis adaptation and survival in macrophages. *Proc Natl Acad Sci U S A*, 102(23), 8327-32 (2005) doi:10.1073/pnas.0503272102

7. S. E. Haydel, V. Malhotra, G. L. Cornelison and J. E. Clark-Curtiss: The prrAB two-component system is essential for Mycobacterium tuberculosis viability and is induced under nitrogen-limiting conditions. *J Bacteriol*, 194(2), 354-61 (2012) doi:10.1128/JB.06258-11

8. P. Sander, K. G. Papavinasasundaram, T. Dick, E. Stavropoulos, K. Ellrott, B. Springer, M. J. Colston and E. C. Bottger: Mycobacterium bovis BCG recA deletion mutant shows increased susceptibility to DNA-damaging agents but wild-type survival in a mouse infection model. *Infect Immun*, 69(6), 3562-8 (2001) doi:10.1128/IAI.69.6.3562-3568.2001

9. A. C. Brown and T. Parish: Dxr is essential in Mycobacterium tuberculosis and fosmidomycin resistance is due to a lack of uptake. *Bmc Microbiology*, 8 (2008) doi:Artn 78

10.1186/1471-2180-8-78

10. A. S. Fivian-Hughes, J. Houghton and E. O. Davis: Mycobacterium tuberculosis thymidylate synthase gene thyX is essential and potentially bifunctional, while thyA deletion confers resistance to p-aminosalicylic acid (vol 158, pg 308, 2012). *Microbiology-Sgm*, 158, 1388-1388 (2012) doi:10.1099/mic.0.X00002-0

11. S. Morayya, D. Awasthy, R. Yadav, A. Ambady and U. Sharma: Revisiting the essentiality of glutamate racemase in Mycobacterium tuberculosis. *Gene*, 555(2), 269-76 (2015) doi:10.1016/j.gene.2014.11.017

12. J. M. Curry, R. Whalan, D. M. Hunt, K. Gohil, M. Strom, L. Rickman, M. J. Colston, S. J. Smerdon and R. S. Buxton: An ABC transporter containing a forkhead-associated domain interacts with a serine-threonine protein kinase and is required for growth of Mycobacterium tuberculosis in mice. *Infect Immun*, 73(8), 4471-7 (2005) doi:10.1128/IAI.73.8.4471-4477.2005

13. S. Gandotra, M. B. Lebron and S. Ehrt: The Mycobacterium tuberculosis proteasome active site threonine is essential for persistence yet dispensable for replication and resistance to nitric oxide. *PLoS Pathog*, 6(8), e1001040 (2010) doi:10.1371/journal.ppat.1001040

14. J. C. Evans, C. Trujillo, Z. Wang, H. Eoh, S. Ehrt, D. Schnappinger, H. I. Boshoff, K. Y. Rhee, C. E. Barry, 3rd and V. Mizrahi: Validation of CoaBC as a Bactericidal Target in the Coenzyme A Pathway of Mycobacterium tuberculosis. *ACS Infect Dis*, 2(12), 958-968 (2016) doi:10.1021/acsinfecdis.6b00150

15. V. Singh, S. Donini, A. Pacitto, C. Sala, R. C. Hartkoorn, N. Dhar, G. Keri, D. B. Ascher, G. Mondesert, A. Vocat, A. Lupien, R. Sommer, H. Vermet, S. Lagrange, J. Buechler, D. F. Warner, J. D. McKinney, J. Pato, S. T. Cole, T. L. Blundell, M. Rizzi and V. Mizrahi: The Inosine Monophosphate Dehydrogenase, GuaB2, Is a Vulnerable New Bactericidal Drug Target for Tuberculosis. *ACS Infect Dis*, 3(1), 5-17 (2017) doi:10.1021/acsinfecdis.6b00102

16. G. S. Kolly, C. Sala, A. Vocat and S. T. Cole: Assessing essentiality of transketolase in Mycobacterium tuberculosis using an inducible protein degradation system. *FEMS Microbiol Lett*, 358(1), 30-5 (2014) doi:10.1111/1574-6968.12536
